# Rapid and Effective Neuronal Conversion of Human Glioblastoma In Vitro and In Vivo Using Potent Small Molecules

**DOI:** 10.1111/cpr.70013

**Published:** 2025-03-11

**Authors:** Ya'nan Hu, Jinming Liu, Jian Tu, Min Yang, Qisheng He, Fei Li, Xiaojing Xu, Zhongqing Ji, Jianwei Xu, Wentao Zhong, Mengwen Yan, Ying Yang, Huanxiang Zhang

**Affiliations:** ^1^ Department of Cell Biology, MOE Key Laboratory of Geriatric Diseases and Immunology Suzhou Medical College of Soochow University Suzhou China; ^2^ Department of Pathology The Second Affiliated Hospital of Soochow University Suzhou China; ^3^ Department of Orthopedics The Second Affiliated Hospital of Soochow University Suzhou China; ^4^ Department of Pharmacology, School of Basic Medicine Guizhou Medical University Guiyang China

**Keywords:** CEND1, differentiation therapy, glioblastoma, neuron‐like cells, small molecules

## Abstract

Exploring effective, prompt and universally applicable approaches for inducing the differentiation of glioblastoma (GBM) into terminally differentiated cells, such as astrocytes or neurons that cease cell division, is pivotal for the success of GBM differentiation therapy. In this study, a neuronal‐specific promoter–reporter system was employed to screen small molecules that promote neural differentiation. The cocktail YFSS, consisting of Y27632, Forskolin, SB431542 and SP600125, which selectively targets the ROCK, cAMP, TGF‐β and JNK signalling pathways, respectively, was found to effectively trigger differentiation in human GBM cells. This process yielded neuron‐like cells within 7 days, inhibited GBM cell proliferation and reduced malignancy traits, such as stemness, migratory and invasive capabilities. Transcriptome sequencing revealed the pathways altered by YFSS, shedding light on its dual role in halting cell proliferation and initiating neuronal differentiation. A notable increase in CEND1 expression, a key molecule in cell cycle and neuronal differentiation regulation, was observed during differentiation. However, CEND1 alone could not replicate YFSS's high conversion efficiency and its depletion reduced the differentiation and restored proliferation of the GBM cells. In vivo, prolonged and localised YFSS application significantly curtailed tumour growth and extended survival in patient‐derived xenograft mice models. In summary, our findings reveal that the small‐molecule cocktail YFSS is an effective means for inducing neuronal differentiation in GBM cells, representing a novel and promising pathway for the advancement of GBM treatment.

## Graphical abstract

A potent combination of small molecules YFSS (Y27632, Forskolin, SB431542, SP600125) that were screened through a neuronal‐specific promoter–reporter system *TUBB3*::mCherry effectively induces neuronal‐GBM conversion in vitro and in vivo, unveiling molecular mechanisms that offer a theoretical basis and a potential therapeutic strategy for treating GBM.

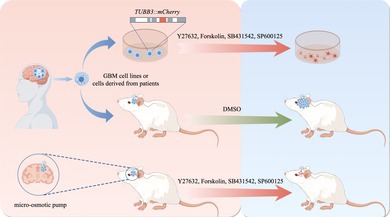

## 1 Introduction

Glioma is the most common primary malignant tumour of the central nervous system and is known for being particularly deadly. Glioblastoma (GBM), the most aggressive type of glioma, is classified as grade IV by the World Health Organization [1]. This type of tumour grows quickly and is highly invasive, making traditional treatments like surgery, radiation and chemotherapy typically ineffective in curing it. Patients with GBM have a median survival time of less than 15 months [2]. Currently, one promising method for treating GBM is to induce terminal differentiation in these cells, resulting in irreversible post‐mitotic arrest. This approach, which can be biological or chemical, does not target abnormal genes but reverses malignant behaviours like proliferation and invasion [3, 4, 5]. This strategy was first proven effective in treating leukaemia, where agents like all‐trans retinoic acid (ATRA) and arsenic trioxide successfully induced cellular differentiation and halted malignancy [6, 7]. However, these differentiation inducers, effective in haematological cancers, are less successful with GBM. The main issue is their limited ability to sufficiently differentiate GBM cells, contributing to poor prognosis and recurrence. Therefore, it is crucial to find a differentiation protocol that not only effectively induces GBM differentiation but also suppresses tumour growth. Since growth factors can promote dedifferentiation in various contexts [8], it is important to investigate if GBM maintains effective differentiation in vivo, as observed in vitro. In contrast to the utilisation of transcription factors or miRNA, small molecule‐induced reprogramming offers several advantages. It circumvents the need for genome insertion of DNA sequences, possesses cellular permeability and non‐immunogenicity, facilitates ease of synthesis, preservation, standardisation and operation, incurs low costs, exhibits rapid biological effects and allows for precise control through varying concentrations and combinations [9, 10, 11]. There is evidence showing that a combination of Forskolin and CHIR99021 can effectively differentiate rat glioma cells into neuron‐like cells and inhibit tumour growth in the spinal cord [12]. Additionally, human GBM cells can be reprogrammed into immature neuron‐like cells using this combination with the addition of ISX9, I‐BET151 and DAPT [13]. Epigenetic reprogramming with histone deacetylase inhibitors enhances cAMP signalling‐induced differentiation in GBM, leading to tumour growth inhibition and extended survival in animal models [14]. Also, using kinase inhibitors targeting ROCK and mTOR concurrently can reprogram human GBM cells into neurons and suppress GBM growth effectively in vivo [15].

CEND1, a neuron‐specific protein, plays a pivotal role in the initial development of the nervous system by coordinating the cessation of the cell cycle in neural progenitor cells and their transformation into neurons [16, 17, 18]. Increasing the expression of CEND1, especially when combined with Neurog2, significantly enhances the conversion of astrocytes into neurons [19, 20]. Moreover, the level of CEND1 expression is closely linked to the survival prognosis of patients with GBM. Manipulating CEND1 levels can significantly impact GBM cell behaviour; reducing CEND1 expression stimulates GBM cell migration and infiltration, while increasing it has the opposite effect [21].

In this study, we identified a potent combination of small molecules—YFSS (Y27632, Forskolin, SB431542, SP600125)—that effectively and rapidly induces the differentiation of GBM cells into mature neuron‐like cells, specifically those positive for tyrosine hydroxylase (TH). This differentiation process is marked by a significant decrease in cell proliferation and cell cycle arrest. Additionally, the malignant traits of GBM cells, such as stemness, migration and invasion, are markedly reduced. Through transcriptional analysis, we have identified several downstream molecules of interest, including CEND1, which is confirmed to be instrumental in the neuronal differentiation of GBM cells induced by YFSS. Remarkably, YFSS shows high efficacy in differentiating patient‐derived GBM cells (PDGCs) and its sustained release in vivo significantly promotes GBM cell differentiation. This leads to a considerable reduction in tumour growth in patient‐derived xenograft (PDX) mouse brains and notably extends their survival duration.

## 2 Materials and Methods

### 2.1 Human GBM Cell Culture and Neural Conversion

Human GBM cell lines (U87, U251, U118, Ln229, Snb19 and T98G) were originally obtained from the American Type Culture Collection (ATCC) and cultured in Dulbecco's modified Eagle's medium (DMEM, Gibco), 10% foetal bovine serum (Gibco) and 1% penicillin–streptomycin (Sigma‐Aldrich). Three human GBM specimens were obtained from patients with written informed consent at the Department of Neurosurgery, the Second Affiliated Hospital of Soochow University. The study protocol was approved by the Ethics Committee of Soochow University. Isolation, propagation and characterisation of GBM primary cultures were performed as previously described [22].

For neural induction, GBM cells were treated with 5 μM Y27632, 50 μM Forskolin, 2 μM SB431542 and 10 μM SP600125 (Selleck) in neural differentiation media (1:1 mixture of DMEM/F12 and neurobasal media, 0.5% N2, 1% B27, 20 ng/mL BDNF, 20 ng/mL NT3, 1% GlutaMax and 1% penicillin/streptomycin) for 1, 3, 5 or 7 days. Cells treated with neural differentiation media without small molecules were used as Control or 0 day.

### 2.2 Lentiviral Preparation and Transduction

The *TUBB3* (NM_001197181.2) promoter followed by fluorescent protein mCherry was ligated into a lentivirus vector to indicate the transcription of *TUBB3* and neuronal differentiation efficiency. The lentivirus vectors carrying CEND1 (NM_016564.4) specific shRNA (CEND1‐shRNA‐1 or CEND1‐shRNA‐2) and its negative control (Scramble), YAP1(NM_006106) specific shRNA YAP1‐shRNA (TRCN0000107268) and its negative control (Scramble) were synthesised by Genscript Corporation (Nanjing, China). The sequences are provided in Table S1. The lentivirus expressing EGFP and luciferase was used to label GBM cells for the tumour growth assay in vivo. A detailed description of the preparation and cell transduction of lentivirus was previously reported [23]. In brief, a three‐plasmid expression system was co‐transfected into HEK293T to produce lentiviral particles. The purified and condensed lentivirus infected the target cells (U87, U251 and PDGCs) at a multiplicity of infection (MOI) of 5. After induction by small molecules, the efficiency of promoter activation in neural differentiated GBM cells was detected by a confocal microscope (Leica).

### 2.3 Cell Cycle Arrest

The distribution of cell cycle phases was determined by propidium iodide staining and examined by flow cytometry. In brief, cells (1.0 × 10^6^) were collected by trypsin digestion, washed with cold PBS and fixed with 70% ethanol overnight at −20°C. Following centrifugation, cells were incubated in PBS with propidium iodide (50 μg/mL) (Sigma‐Aldrich) and RNase (1.0 mg/mL) (Sigma‐Aldrich) in the dark at 37°C for 30 min and analysed by fluorescence‐activated cell sorter (FACS; BD). Visualisation and analysis of flow cytometry data were performed using the FlowJo software (FlowJo 10.6.2) and ModFit LT software (ModFit LT 5.0).

### 2.4 Western Blot Analysis

Western blot analysis was performed as previously described [24]. The GBM cells were lysed and homogenised in RIPA buffer with protease inhibitor and phosphatase inhibitor. Protein samples (25 μg) were loaded on a 10% or 15% SDS‐PAGE gel. The gel was running and transferred to PVDF membranes (Millipore). The membrane was blocked for 1 h at room temperature with 5% BSA in TBST buffer and incubated at 4°C overnight with indicated primary antibodies against TUBB3 (BioLegend, 1:1000), GAPDH (Cell Signalling Technologies, CST, 1:1000), CyclinD1 (CST, 1:1000), CDK2 (CST, 1:1000), p27KIP1 (CST, 1:1000), p21CIP1 (CST, 1:1000), CEND1 (Abcam, 1:10,000) and YAP1 (CST, 1:1000). HRP‐conjugated secondary antibody (anti‐mouse: Invitrogen, 1:10,000 or anti‐rabbit: Invitrogen, 1:10,000) was incubated for 1 h at room temperature and the protein bands were visualised by using chemiluminescence reagents (Millipore). Relative expression was normalised to GAPDH and quantified by densitometry using ImageJ software.

### 2.5 Cell Proliferation Assay

Cell proliferation assay was performed using an EdU assay kit (Beyotime). Briefly, 10 μM EdU was incorporated into cells for 2 h at 37°C. Then the cells were fixed, permeabilised and incubated with Click reaction reagent for 30 min. After staining for nucleic acids with Hoechst33258 (Sigma‐Aldrich), images were obtained by using a confocal microscope (Leica).

### 2.6 Immunofluorescence

Immunofluorescence staining was performed as previously described [23]. Briefly, GBM cells or sections were fixed in cold 4% paraformaldehyde in 0.1 M PBS (pH 7.2) for 30 min and permeabilised with PHT solution (3% BSA and 0.1% Triton X‐100 in PBS) for 1 h at room temperature. Primary antibodies against TUBB3 (BioLegend, dilution 1:500), MAP2 (CST, dilution 1:50), PSD95 (CST, dilution 1:200), SYN1 (CST, dilution 1:200), Ki‐67 (CST, dilution 1:400), NEUN (Abcam, dilution 1:200) and TH (Abcam, dilution 1:200) were used and visualised using Alexa Fluor 594 goat anti‐mouse IgG (H + L) (Invitrogen, dilution 1:500) or anti‐rabbit IgG (H + L) (Invitrogen, dilution 1:500). Cell nuclei were counterstained with Hoechst33258 (Sigma‐Aldrich). Confocal laser scanning (Leica) was used to detect the fluorescence signals.

### 2.7 Calcium Imaging

Calcium imaging and data analysis were performed by Leica Microsystems LAS AF (Leica AF6000 microscopy). GBM cells (U87 and U251) differentiated by YFSS for 7 days were loaded with 2 μM Fluo‐4 AM (Beyotime) for 30 min at 37°C in 5% CO_2_. Fluo‐4 AM was added to Tyrode's solution (128 mM NaCl, 2 mM KCl, 2 mM CaCl_2_, 2 mM MgCl_2_, 30 mM glucose and 25 mM HEPES). Calcium flux was monitored for 90 s, with a 200‐ms exposure time and a 2‐s interval between exposures. For KCl stimulation experiments, a final concentration of 25 mM was added to confirm the neural identity of responsive cells. Calcium responses were calculated as the change in fluorescence (Δ*F*) over the initial fluorescence (*F*0) quantified by using ImageJ software.

### 2.8 Tumour Sphere Formation Assay

To assess the clonogenicity of GBM cells after neural induction, 100 cells were seeded per well in 96‐well clear round‐bottom ultra‐low‐attachment microplates (Corning) in neural progenitor medium (neurobasal, 1% N2, 1% B27, 10 ng/mL EGF and 20 ng/mL bFGF). Tumour sphere numbers and areas were counted and scored using the ImageJ programme.

### 2.9 In Vitro Limiting Dilution Assay

For tumoursphere‐formation assays, an in vitro limiting dilution assay (LDA) was performed. Briefly, cells were plated in non‐adherent 96‐well plates with diluted cell numbers ranging from 50 to 1 cell/well (−50, 20, 10, 5, 2, 1) with eight replicates per condition in 100 μL serum‐free DMEM/F12 medium supplemented with 20 ng/mL bFGF, 10 ng/mL EGF, 2% B27 (Invitrogen) and 10 μg/mL insulin. The presence of spheres in each well was recorded 7 days after plating. GBM sphere‐forming frequencies were evaluated using extreme limiting dilution analysis (http://bioinf.wehi.edu.au/software/elda/).

### 2.10 qRT‐PCR and RNA‐Seq

Total RNA was isolated using Trizol reagent (Invitrogen) according to the user guide for qRT‐PCR and RNA‐seq analysis. For qRT‐PCR, total RNA was reverse transcribed using the Maxima First Strand cDNA Synthesis Kit for RT‐qPCR (Thermo Fisher Scientific) according to the supplier's recommended procedure. qRT‐PCR detection was performed with SYBR Premix Ex Taq (Takara Bio) using the primers listed in Table S1. All samples were processed in triplicate. The average value was used for the measurements and the results were normalised to the expression of GAPDH.

For RNA‐seq, total RNA was extracted and the eukaryotic mRNA was enriched by Oligo (dT) beads. Then the enriched mRNA was fragmented into short fragments using fragmentation buffer and reversely transcribed into cDNA by using NEBNext Ultra RNA Library Prep Kit for Illumina (New England Biolabs, NEB). The purified double‐stranded cDNA fragments were end‐repaired and A base was added and ligated to Illumina sequencing adapters. The ligation reaction was purified with the AMPure XP Beads and amplified by PCR. The resulting cDNA library was sequenced using Illumina Novaseq6000 by Gene Denovo Biotechnology Co. (Guangzhou, China). The RNA‐Seq data were then used in Gene Set Enrichment Analysis (GSEA, v2.2.4) and analysed by weighted gene co‐expression network analysis (WGCNA) with WGCNA R package. For differentially expressed genes from RNA‐seq data, gene ontology (GO) and KEGG pathway analysis were performed using DAVID (https://david.ncifcrf.gov). The transcriptome data generated in the paper are deposited in the NCBI Gene Expression Omnibus (GEO). The accession number is GSE247893.

### 2.11 Migration and Invasion Assays

The migration and invasion of GBM cells or neural differentiated GBM cells were conducted with Transwell chambers. Cells were seeded at a density of 5 × 10^4^ cells per 100 μL in neural differentiation media with or without small molecules onto 24‐well plates with Transwell inserts of 8 μm pore size (Corning) for migration assay or onto Transwell upper chambers coated with Matrigel (BD Biosciences) for invasion assay and the lower chamber was filled with the same media as the upper. After 24‐h incubation, non‐migrated or non‐invaded GBM cells were scraped off. Cells on the bottom surface were fixed with 4% paraformaldehyde for 10 min and stained with 0.4% crystal violet for 30 min. The numbers of migrated and invaded cells were counted from five randomly chosen fields using ImageJ.

### 2.12 In Vivo Xenograft Tumour Models

Differentiation therapy of GBM was evaluated in vivo using an intracranial GBM xenograft model. In accordance with Chinese law, animals were used after approval from the Soochow University Veterinary Authority. The study protocol was approved by the Ethics Committee of Soochow University. Prior to the implantation procedure, the mice were anaesthetised with an intraperitoneal (i.p.) injection of ketamine (100 mg/kg). U87 or human primary GBM cells expressing luciferase (2 × 10^5^ cells per mouse) were suspended in 5 μL PBS and implanted into the brain of 8‐week‐old immunodeficient NOD‐SCID mice using a stereotactic device (Kopf Instruments) (coordinates: 2 mm posterior, 2 mm lateral to the bregma and 2.5 mm depth from the dura) [25].

At 7 days post‐implantation, 100 μL of artificial cerebrospinal fluid with or without small molecules (15 μM Y27632, 150 μM Forskolin, 6 μM SB431542 and 30 μM SP600125) was sustained‐release into orthotopic tumour tissues by an osmotic mini‐pump (RWD Life Science, China) for 14 days. In brief, mice were deeply anaesthetised and placed into a stereotaxic apparatus. After blunt dissection, a subcutaneous pocket was made in the mid‐scapular region to insert the osmotic mini‐pump. The pump was connected via a catheter to a microcannula for brain infusion, which was stereotaxically implanted at the same location as the transplanted cells. These mini‐pumps delivered at a flow rate of 0.25 μL/h during the 14‐day treatment period. After the surgery, mice were sutured and kept warm until they fully recovered.

Luciferase activity was measured to monitor tumour volume weekly by detecting the bioluminescence using the PerkinElmer IVIS Spectrum. All mice were euthanized when they reached a moribund condition. Their brains were removed, embedded, sectioned on a cryostat in 20 μm thickness and stained with H&E or immunofluorescence. Survival data of the xenograft tumour model were compared using the Kaplan–Meier method with the log‐rank test (*n* = 8). All experiments were approved by the institutional animal care and use committees.

### 2.13 Statistical Analysis

Data are presented as mean ± SEM. Statistical analysis was performed with Student's *t* test or one‐way analysis of variance (ANOVA) followed by the Bonferroni multiple comparisons test. *,^#^ *p* < 0.05 and **,^##^ *p* < 0.01, were considered statistically significant. All experiments were independently repeated at least three times.

## 3 Results

### 3.1 Efficient Induction of Neuronal Differentiation of GBM Cells Using Small Molecule Cocktails

In our previous study, we successfully reprogrammed fibroblasts into neurons using small molecules, showing promise for spinal cord injury repair [23]. To find small molecules that efficiently differentiate human GBM cells into neurons, we developed a *TUBB3* promoter–reporter system and established a U87‐*TUBB3*::mCherry cell line, enabling TUBB3 monitoring, a neuron‐specific marker. We screened various small molecules known for their roles in neuronal trans‐differentiation, including Y27632, SB431542, CHIR99021, LDN193189, SP600125, DAPT, SU5402, VPA, Forskolin, ISX9, I‐BET151, among others, using U87‐*TUBB3*::mCherry cells. The cocktail YFSS (Y27632, Forskolin, SB431542, SP600125) was found to effectively induce neuron differentiation in U87 cells, with an efficiency of 92.4% ± 0.9%, as indicated by mCherry expression. Omitting any YFSS component markedly reduced differentiation efficiency (Figure S1A,B). In vitro differentiation of U87 and U251 cells, achieved with a YFSS‐supplemented neuronal induction medium, led to significant morphological changes within 1 day, even as early as 2 h (Movie S1), including the loss of pleomorphic features and the development of elongated neuron‐like structures. These morphological changes further evolved into intricate, neuron‐like processes (Figure S1C), alongside strong TUBB3 expression (Figure S1A).

**FIGURE 1:**
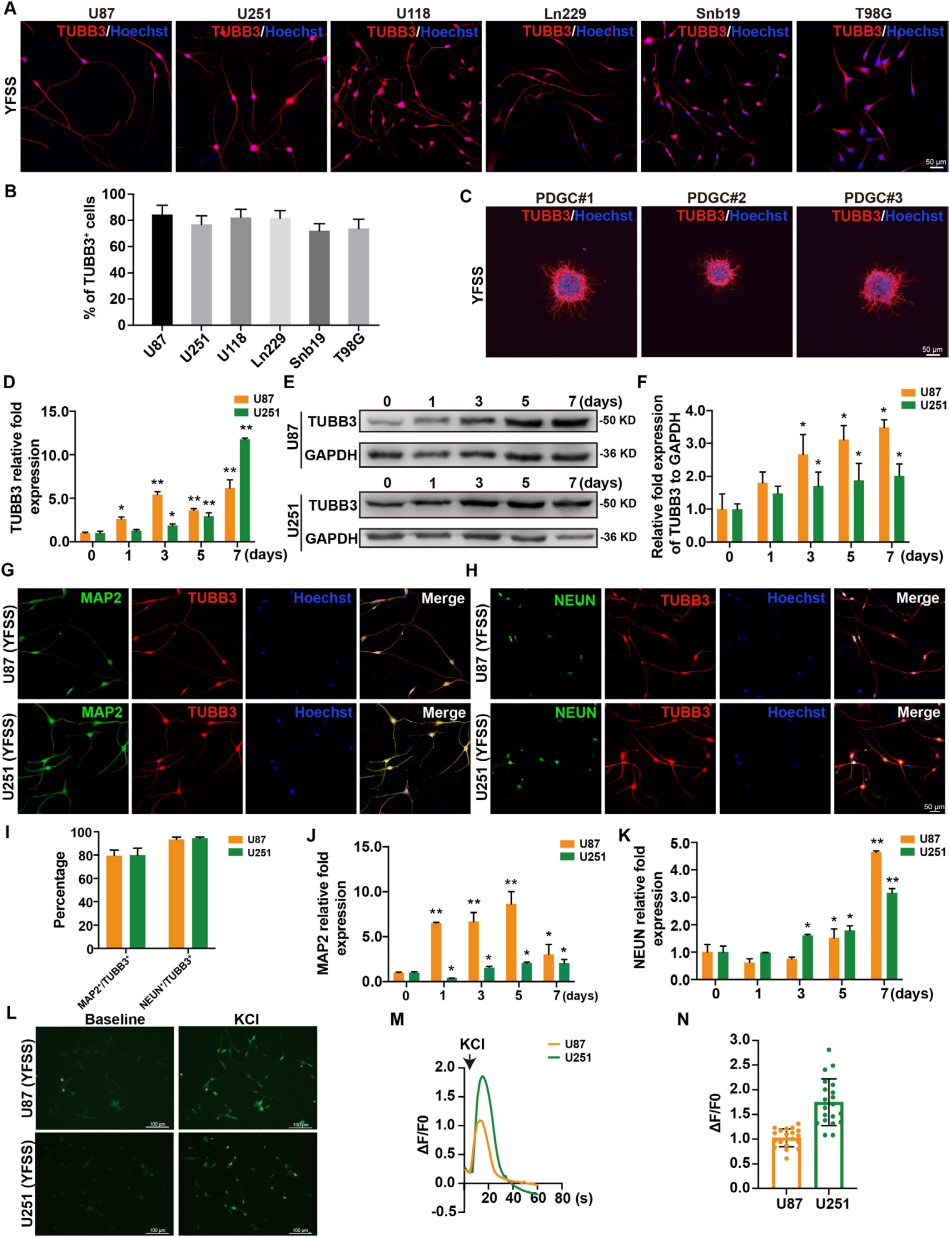
YFSS mediates effective neuronal fate change of GBM cells. (A, B) GBM cell lines (U87, U251, U118, Ln229, Snb19 and T98G) were induced to differentiate by YFSS for 3 days and stained for neuronal marker TUBB3 and labelled with Hoechst33258 (A), quantification of percentages of TUBB3^+^ cells (B), Bar = 50 μm. (C) Patient‐derived GBM cells (PDGC) were induced to differentiate by YFSS for 7 days and stained for neuronal marker TUBB3 and labelled with Hoechst33258, Bar = 50 μm. (D–F) Relative expression of TUBB3 in YFSS‐induced GBM cells at different stages (0, 1, 3, 5 and 7 days) by qRT‐PCR analysis (D) and Western blot analysis (E, F), *p < 0.05, **p < 0.01. (G–I) Representative images of GBM cells treated with YFSS for 7 days double stained for TUBB3 and MAP2 or NEUN and labelled with Hoechst33258 (G, H), Bar graph percentage of MAP2^+^ or NEUN^+^ in TUBB3 positive cells (I), Bar = 50 μm. (J, K) Relative expression of MAP2 (J) and NEUN (K) in YFSS‐induced GBM cells at different stages (0, 1, 3, 5 and 7 days) by qRT‐PCR analysis, *p < 0.05, **p < 0.01. (L) Representative images of GBM cells treated with YFSS for 7 days loaded with Fluo‐4‐AM to examine the intracellular calcium fluctuations in response to KCl (10 mM), Bar = 100 μm. (M) Calcium responses were calculated as the change in fluorescence (Δ*F*) over the initial fluorescence (*F*0). (N) Δ*F*/*F*0 intensity plot showing the response of individual cells to KCl (*n* = 20).

In our study, TUBB3^+^ cells in GBM cell lines (U87, U251, U118, Ln229, Snb19 and T98G) showed a marked increase to 84.5% ± 7.1%, 76.9% ± 6.5%, 82.3% ± 6.1%, 81.6% ± 6.0%, 72.0% ± 5.3% and 73.9% ± 7.0% respectively, 3 days post‐induction (Figure 1A,B). Considering GBM's heterogeneity and mutation diversity, patient‐derived samples offer a closer representation of its genetic and phenotypic characteristics than traditional cell lines. We isolated cells from resected GBM tissues of three patients and cultured them in neurosphere medium. These cells, once adhered to a PLL‐coated matrix, were subjected to YFSS in a neuronal induction medium for 7 days. The PDGCs formed spheres and underwent neuronal differentiation as indicated by increased TUBB3 fluorescence intensity (Figure 1C). A time‐course analysis revealed a steady rise in TUBB3 expression at both mRNA and protein levels during differentiation, with a significant increase after 3 days in U87 and U251 (Figure 1D–F). Additionally, MAP2 presence was notable, with rates of 79.6% ± 4.6% in U87 and 80.0% ± 6.0% in U251, as illustrated in Figure 1G,I. Similarly, MAP2 expression began to increase on the first and third days of differentiation in U87 and U251, respectively, and remained at a high level thereafter (Figure 1J). After a 7‐day YFSS induction, cells also demonstrated pronounced NEUN staining, with U87 and U251 showing 93.4% ± 2.0% and 94.4% ± 1.1% positivity, respectively (Figure 1H,I). Moreover, NEUN expression in U87 and U251 cells significantly increased by Days 5 and 3 of differentiation, respectively, indicating maturation (Figure 1K). Furthermore, synaptic proteins PSD95 and SYN1 also showed positive staining (Figure S1D).

To evaluate the functional properties of the neuron‐like cells derived from differentiation, we conducted calcium imaging after 7 days of differentiation. Both differentiated U87 and U251 cells, when treated with potassium chloride (KCl), displayed the characteristic neuronal calcium transient response. This response was marked by a rapid and significant increase in intracellular calcium levels, as shown in Figure 1L–N and Movies S2 and S3. These findings suggest that the differentiated cells have acquired the physiological function of neurons. Consequently, these results demonstrate that the YFSS small molecule cocktail is effective in inducing the differentiation of human GBM cells into functional neuron‐like cells.

### 3.2 YFSS‐Mediated Neuronal Conversion of GBM Cells Leads to Decreased Cell Proliferation and Promotes Cell Cycle

**FIGURE 2:**
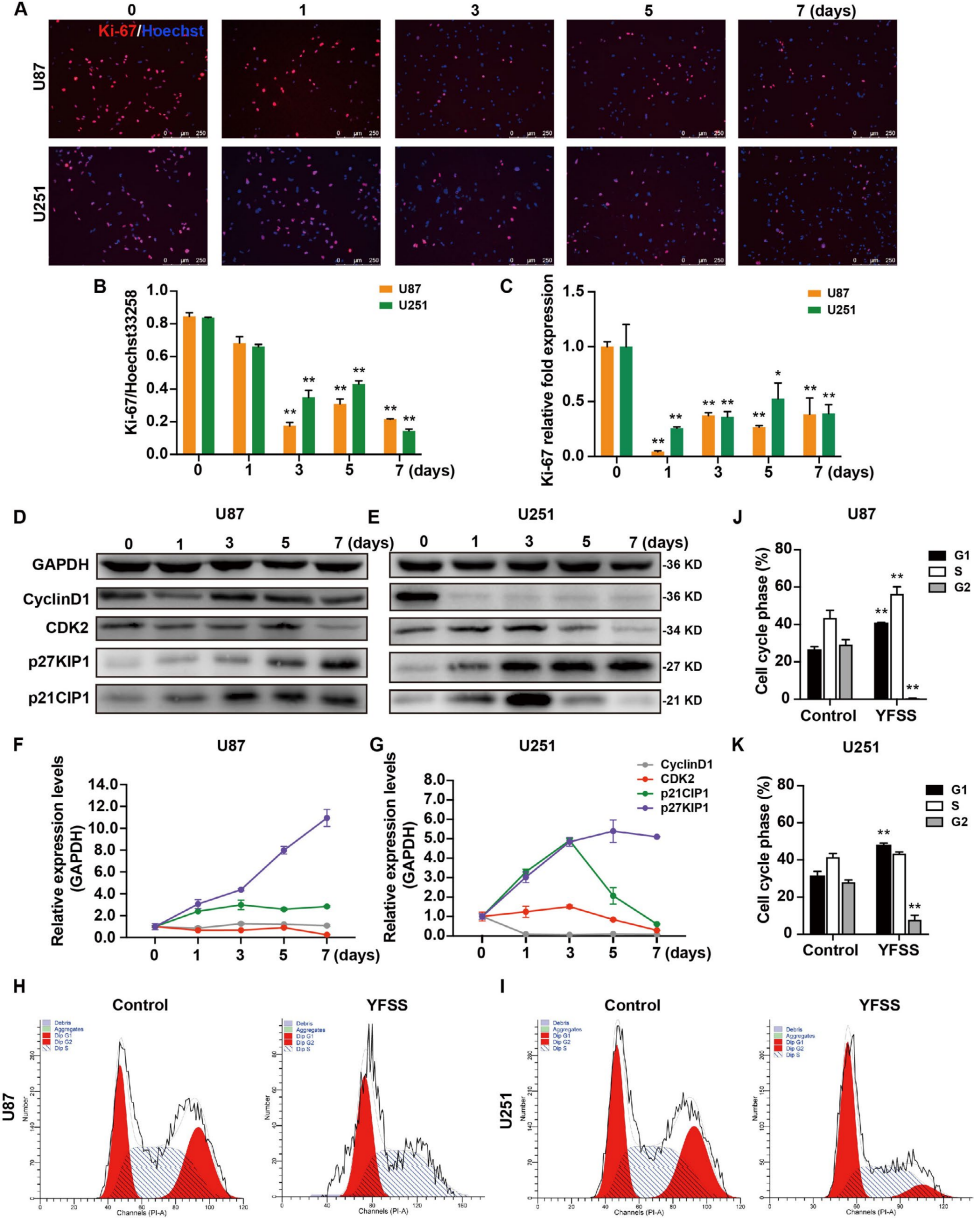
YFSS Inhibits cell proliferation and leads to cell cycle arrest. (A, B) Representative images of YFSS‐induced GBM cells at different stages (0, 1, 3, 5 and 7 days) stained for Ki‐67 and labelled with Hoechst33258 (A), quantification of percentages of Ki‐67 positive cells (B), Bar = 250 μm. (C) qRT‐PCR of Ki‐67 expression in YFSS‐induced GBM cells at different stages (0, 1, 3, 5 and 7 days), *p < 0.05, **p < 0.01. (D–G) Relative expression of CyclinD1, CDK2, p27KIP1 and p21CIP1 in YFSS‐induced GBM cells at different stages (0, 1, 3, 5 and 7 days) was quantified by Western blot analysis. (H–K) Cell cycle analysis through PI staining and following flow cytometry for the cells of undifferentiated (Control) and cells treated with YFSS for 7 days (YFSS) (H, I), the quantitative measurement of cell cycle phase (J, K), **p < 0.01.

To assess the proliferation of GBM cells (U87 and U251) during neuronal differentiation, we examined Ki‐67 expression at different stages (0, 1, 3, 5 and 7 days). Immunofluorescence staining showed a marked decrease in Ki‐67‐positive cells from Day 3 of differentiation (Figure 2A,B), with a significant reduction in Ki‐67 mRNA levels observed from Day 1 (Figure 2C). This suggests a notable impediment in cell proliferation during differentiation. Further analysis using EdU incorporation over 7 days revealed a substantial decline in proliferative activity post‐YFSS treatment, with EdU positivity decreasing from 33.4% ± 3.4% to 4.4% ± 0.5% in U87 and from 45.4% ± 1.2% to 16.4% ± 1.6% in U251 (Figure S2A,B). These results clearly indicate a significant reduction in the proliferation of GBM cells as they differentiate into neuron‐like cells.

To understand the role of cell cycle regulation in GBM cell proliferation during differentiation, we analysed U87 cells at various differentiation stages (0, 1, 3, 5 and 7 days). Transcriptome microarray analysis showed a consistent decrease in cyclin proteins (Cyclins) and cyclin‐dependent kinases (CDKs) expression, including CyclinA2, B1, B2, D1, E1, E2 and CDK1, 2, 4, 6, 7 and 8. In contrast, CDK inhibitors (CKIs) like p21CIP1 and p27KIP1 increased throughout differentiation (Figure S3A). KEGG analysis using GSEA confirmed significant downregulation of cell cycle and DNA replication pathways in differentiated cells (Figure S2C,D).

qRT‐PCR analysis validated these findings, revealing a significant reduction in Cyclins and CDKs expression from the onset of differentiation, while CKIs p21CIP1 and p27KIP1 showed increased expression (Figure S3B–E). CyclinD1 protein levels mirrored mRNA levels, decreasing initially in U87 cells and then returning to baseline after 3 days, while remaining consistently low in U251 cells. CDK2 expression patterns varied temporally, with initial decreases followed by fluctuations in both cell lines. The expression levels of p21CIP1 and p27KIP1, at the protein levels, exhibited a consistent increase throughout the differentiation process of U87 cells. In U251 cells, the expression of both p21CIP1 and p27KIP1 also increased, with the former reaching its maximum level at Day 3 and the latter at Day 5 (Figure 2D–G).

Flow cytometry analysis revealed that after 7 days of YFSS treatment, there was a marked increase in the G1 phase and a decrease in the G2 phase in both U87 and U251 cells. Notably, U87 cells also showed an increase in the S phase (Figure 2H–K). These findings strongly suggest that YFSS treatment impedes cell proliferation and induces a cell cycle arrest in G1 and S phases during GBM cell differentiation into neurons.

### 3.3 YFSS Suppresses Stemness and Reduces the Migratory and Invasive Capabilities of GBM Cells

**FIGURE 3:**
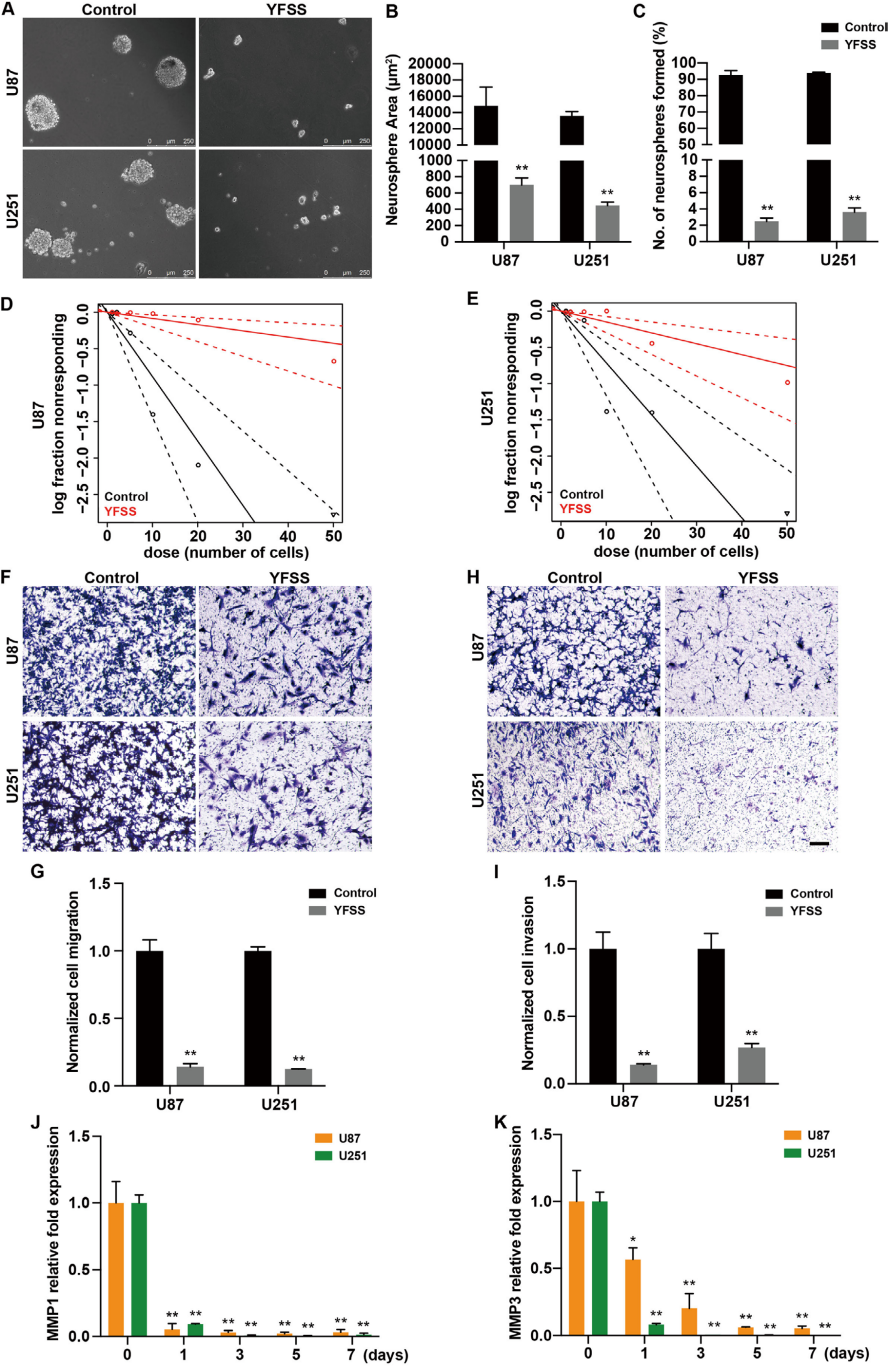
YFSS reverses the malignant phenotype of GBM. (A–C) Representative images from tumour sphere formation assay in GBM cell when treated with DMSO (Control) or YFSS for 7 days (YFSS) (A), and statistical analysis of the average area of tumour spheres (B) and the number of tumour spheres with a diameter larger than 100 μm (C), **p < 0.01, Bar = 250 μm. (D, E) Line diagram depicts a decrease in self‐renewal capacity upon YFSS induction, as analysed under serum‐free conditions using limiting dilution assay. (F–I) Representative images from Transwell assay in GBM cells (Control) or cells treated with YFSS for 7 days (YFSS) during the migration (F, G) and invasion (H, I), **p < 0.01, Bar = 50 μm. (J, K) Relative expression of invasion‐related proteins MMP1 (J) and MMP3 (K) was quantified by qRT‐PCR in YFSS‐induced GBM cells at different stages (0, 1, 3, 5 and 7 days), *p < 0.05, **p < 0.01.

The diminished proliferation and cell cycle exit observed in GBM cells undergoing neuronal differentiation by YFSS led us to hypothesise that stemness, migration and invasion could be similarly affected. When U87 and U251 cells were cultured in neurosphere medium for 7 days, YFSS significantly inhibited spheroidisation ability, as observed in the tumour sphere formation assay (Figure 3A–C) and in vitro LDA (Figure 3D,E).

Migration was assessed using a Transwell assay, which showed a significant reduction in the number of cells that migrated to the lower chamber after 7 days of differentiation, compared to undifferentiated cells (Figure 3F,G). The invasive capacity of both cell lines, measured by a Matrigel invasion assay, also significantly decreased after differentiation (Figure 3H,I). The expression levels of invasion‐associated genes MMP1 and MMP3 declined from the first day of differentiation, suggesting a potent decrease in invasive potential (Figures 3J,K and S4A). The expression of other invasion‐related genes, vascular endothelial growth factor C (VEGFC) and ADAM metallopeptidase with thrombospondin type 1 motif 1 (ADAMTS1) followed a similar downward trend, while tissue inhibitor of metalloproteinase (TIMP) family genes, namely TIMP1, TIMP2 and TIMP4, known as invasion inhibitors, showed increased expression (Figure S4A).

These observations collectively indicate that YFSS not only inhibits tumour sphere formation but also markedly reduces the migration and invasion of GBM cells during neuronal differentiation, contributing to a reversal of the GBM malignant phenotype.

### 3.4 Molecular Mechanism of Differentiation of GBM Into Neuron‐Like Cells Induced by YFSS

**FIGURE 4:**
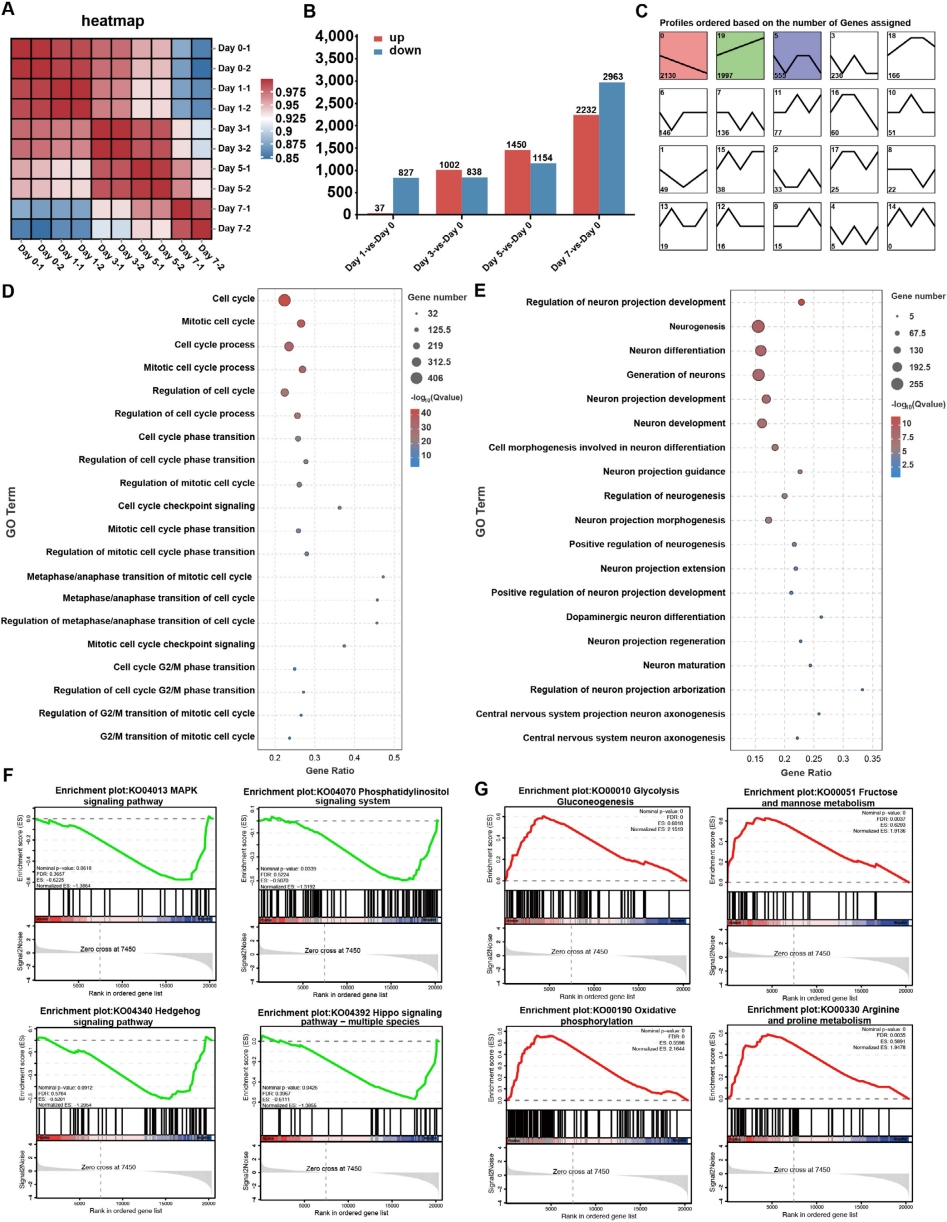
Transcriptome analysis is conducted during YFSS‐induced GBM differentiation towards neurons. (A) Correlation among transcriptomic datasets in YFSS‐induced U87 at different stages (Days 0, 1, 3, 5 and 7). (B) Number of genes that significantly up‐or down‐regulated in U87 at different stages (Days 1, 3, 5 and 7) compared to undifferentiated cells (Day 0) (fold change ≥ 2, FDR ≤ 0.05). (C–E), WGCNA analysis of the genes at different differentiation stages (Days 0, 1, 3, 5 and 7) in U87 and eigengenes expression patterns of 20 modules of WGCNA clustering (C), GO‐BP analysis of the key modules (0, consistent decrease; 19, consistent increase) from WGCNA (D, E). (F, G) GSEA analysis focusing on the differential enrichment of KEGG pathways (Days 1 vs. 0).

To elucidate the molecular basis of YFSS‐induced neuronal differentiation in GBM cells, we analysed gene expression changes in the U87 transcriptome over a 7‐day period. The correlation between the gene expression profiles of differentiated GBM cells and their original state weakened over time, indicating a shift towards a neuronal state (Figure 4A,B). We identified 20 gene expression trends, with three main patterns: a consistent decrease (2130 genes), a consistent increase (1997 genes) and a fluctuating pattern (555 genes) (Figure 4C).

GO enrichment analysis of the downregulated genes highlighted their involvement in cell cycle processes, suggesting a decrease in cell proliferation (Figure 4D). Upregulated genes were significantly enriched in biological processes related to neuronal development, including pathways associated with the generation of dopaminergic neurons, confirmed by the presence of the dopaminergic marker TH positive cells (Figures 4E and S1D). This implies that YFSS may promote the conversion of GBM into dopaminergic neuron‐like cells.

KEGG pathway analysis further indicated that consistently upregulated genes were linked to cell adhesion, axon guidance, glycan biosynthesis, amino acid metabolism and Wnt signalling (Figure S4B). GSEA revealed that dynamic changes in signalling pathways such as MAPK signalling, phosphatidylinositol signalling, Hedgehog signalling and Hippo signalling (Figure 4F) and in metabolism pathways such as glycolysis, gluconeogenesis, fructose, mannose, oxidative phosphorylation, arginine and proline (Figure 4G) were seen as early as Day 1 of differentiation.

To validate the role of Hippo signalling in initiating differentiation, we investigated the effects of YAP1 knockdown (Figure S5A,B), which activates Hippo signalling. This intervention significantly suppressed YFSS‐induced GBM differentiation, as evidenced by a marked reduction of TUBB3 expression (Figure S5C), alongside an increase in Ki‐67‐positive cells (Figure S5D) and elevated expression of the proliferation‐associated CyclinD1 (Figure S5E,F). In contrast, pharmacological inhibition of Hippo signalling using the small molecule XMU‐MP‐1 had no effect on YFSS‐induced GBM differentiation. Moreover, standalone inhibition of Hippo signalling was insufficient to induce GBM differentiation (Figure S5G). These findings indicate that suppression of Hippo signalling is necessary for YFSS‐induced GBM differentiation, but inhibition of Hippo signalling alone is not sufficient to mimic the effect of YFSS.

Collectively, these results suggest that YFSS triggers a complex network of gene expression changes, leading to the reduction of GBM proliferative capacity and promoting neuronal differentiation.

### 3.5 CEND1 Is Implicated in the Regulatory Mechanisms of Neuronal Differentiation in GBM Cells Induced by YFSS

**FIGURE 5:**
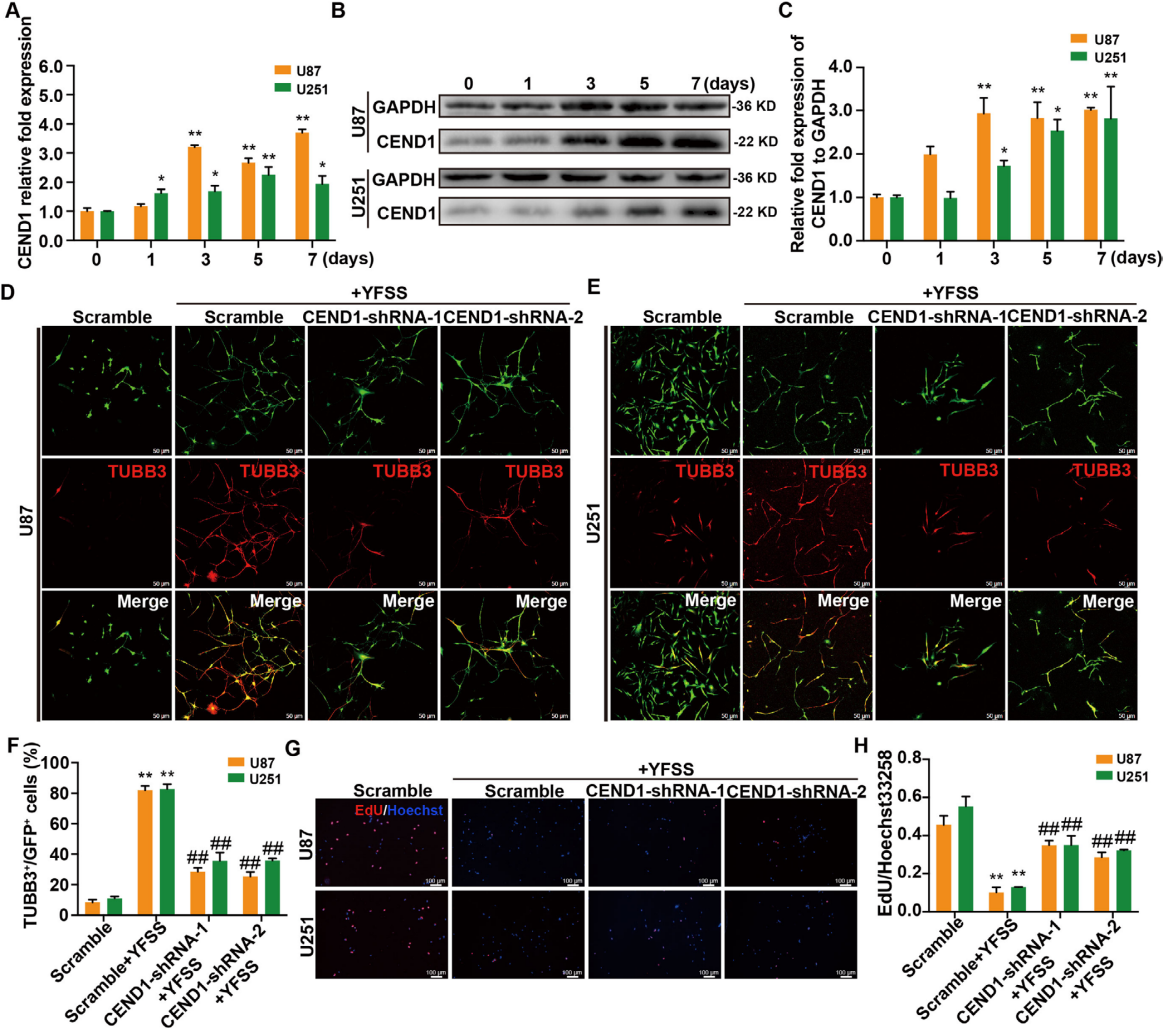
CEND1 plays a key role in YFSS‐induced neuronal‐GBM conversion. (A–C) Relative expression of CEND1 in YFSS‐induced GBM cells at different stages (0, 1, 3, 5 and 7 days) by qRT‐PCR analysis (A) and Western blot analysis (B, C), *p < 0.05, **p < 0.01. (D–F) Representative images of GBM cells treated with control siRNA (Scramble) or siRNA targeting CEND1 (CEND1‐shRNA‐1 or CEND1‐shRNA‐2) induced by YFSS for 7 days and stained for TUBB3 (D, E), and percentage of TUBB3^+^ in GFP positive cells (F), **p < 0.01 versus Scramble; ^##^ *p* < 0.01 versus Scramble + YFSS, Bar = 50 μm. (G, H) Representative images of GBM cells treated with control siRNA (Scramble) or siRNA targeting CEND1 (CEND1‐shRNA‐1 or CEND1‐shRNA‐2) induced by YFSS for 7 days and labelled with EdU and Hoechst33258 (G) and the relative EdU incorporation rate was calculated (H), **p < 0.01 versus Scramble; ^##^ *p* < 0.01 vs. Scramble + YFSS, Bar = 100 μm.

We noted an increase in the expression of molecules involved in cell cycle regulation and neuronal differentiation during YFSS‐induced differentiation of GBM cells into neuron‐like cells. One such molecule, CEND1, a neuron‐specific protein that facilitates cell cycle exit and promotes neuronal differentiation, showed progressively higher expression levels in our U87 transcriptome analysis over a 7‐day period (Figure S6A). This upregulation was confirmed by qRT‐PCR, with a significant expression increase noted at 3 days post‐differentiation, which then remained elevated (Figure 5A). Protein levels of CEND1 mirrored this trend (Figure 5B,C).

To determine the role of CEND1 in this differentiation, we used lentiviruses to reduce its expression in U87 and U251 cells. The knockdown of CEND1, confirmed at both mRNA and protein levels (Figure S6B–E), led to a noticeable decrease in differentiation, as indicated by lower TUBB3 positivity (Figure 5D–F). While CEND1 reduction partly reversed YFSS‐induced suppression of GBM cell proliferation, it was not entirely abrogative (Figure 5G,H). Moreover, forced overexpression of CEND1 alone did not trigger TUBB3 expression, suggesting that additional factors are involved in the YFSS‐induced neuronal differentiation of GBM cells (Figure S6F–K). These findings suggest that while CEND1 plays a key role in this process, it operates within a larger regulatory network.

**FIGURE 6:**
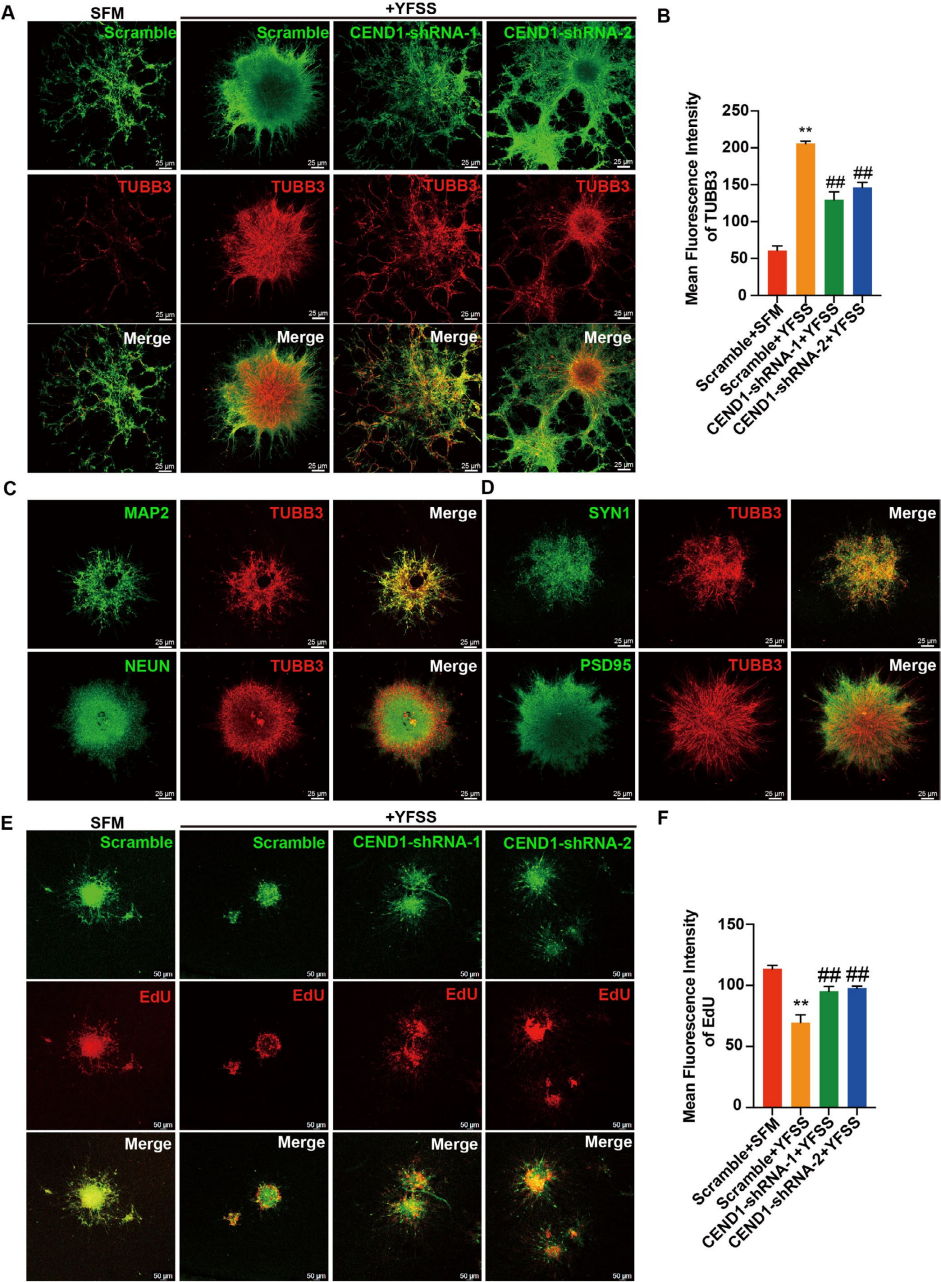
YFSS mediates effective neuronal fate change of PDGC. (A, B) Representative images of PDGC treated with control siRNA (Scramble) or siRNA targeting CEND1 (CEND1‐shRNA‐1 or CEND1‐shRNA‐2) induced by YFSS for 3 days and stained for TUBB3 (A), and the average fluorescence intensity was quantified for each group (B), **p < 0.01 versus Scramble; ^##^ *p* < 0.01 versus Scramble + YFSS, Bar = 25 μm. (C, D) Representative images of PDGC treated with YFSS for 7 days and double stained for TUBB3 and MAP2, NEUN, PSD95 or SYN1, Bar = 25 μm. (E, F) Representative images of PDGC treated with control siRNA (Scramble) or siRNA targeting CEND1 (CEND1‐shRNA‐1 or CEND1‐shRNA‐2) induced by YFSS for 3 days and labelled with EdU (E), and the average fluorescence intensity was quantified for each group (F), **p < 0.01 versus Scramble; ^##^ *p* < 0.01 versus Scramble + YFSS, Bar = 25 μm.

YFSS‐induced neuronal differentiation of PDGC was significantly enhanced, as evidenced by increased TUBB3 fluorescence intensity (Figure 6A,B), co‐localization with mature neuronal markers MAP2, NEUN, SYN1 and PSD95 (Figure 6C,D) and reduced EdU incorporation compared to the control group (Scramble + SFM) (Figure 6E,F), while this phenomenon was partially reversed by CEND1 knockdown (Figure 6A,B,E,F).

Additionally, the migration of PDGC was examined. Without treatment, GBM spheres expanded over time, but YFSS treatment notably inhibited this spread. This effect was somewhat lessened by CEND1 downregulation, highlighting CEND1's role in reducing GBM cell dispersal and migration during differentiation (Figure S7).

These results demonstrate that YFSS not only induces differentiation of PDGC but also implies that CEND1 is a key regulator in this transformative process.

### 3.6 YFSS‐Induced Reprogramming Hinders GBM Tumour Growth in Vivo

To assess the clinical potential of YFSS in vivo, we utilised the U87 and PDGC engineered to express luciferase‐GFP for tracking tumour growth. We established a PDX model and administered YFSS directly to the tumour site via a sustained‐release micro‐osmotic pump. This method allowed for controlled release and facilitated the in vivo differentiation of GBM cells into neurons. Tumour progression was monitored weekly using bioluminescence imaging with the PerkinElmer IVIS Spectrum.

**FIGURE 7:**
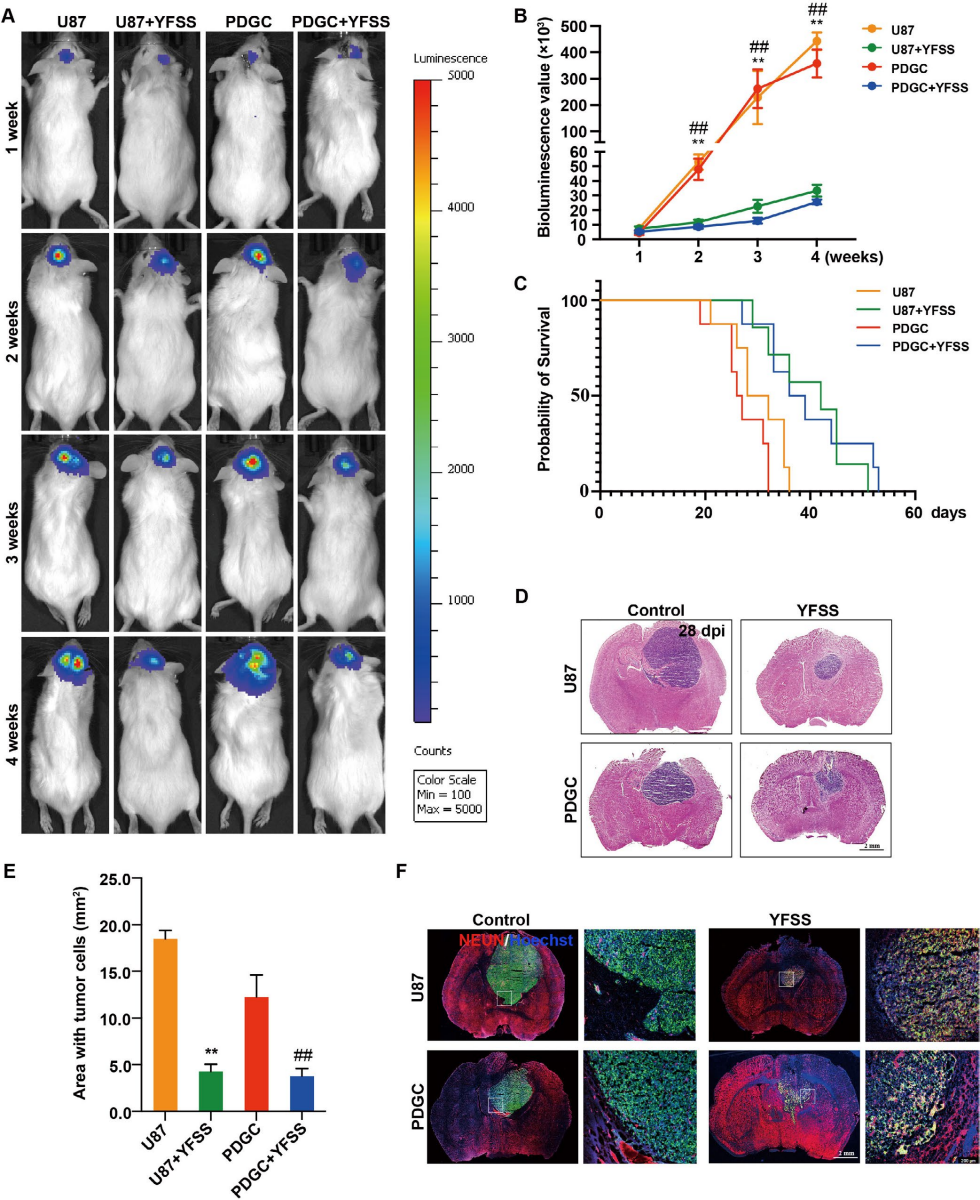
YFSS suppresses tumour development and prolongs mouse survival. (A, B) Representative in vivo bioluminescent images (A) and quantification of bioluminescent signals (B) of brain tumour in mice with indicated treatment at Weeks 1, 2, 3 and 4 after implantation, **p < 0.01 U87 versus U87 + YFSS; ^##^ *p* < 0.05 PDGC versus PDGC + YFSS. (C) Kaplan–Meier plot of survival of mice allografted with U87 or PDGC with or without YFSS sustained release (*n* = 8 per group). (D, E) Representative H&E images (D) and quantification (E) of tumour area of brain sections from mice with indicated treatment for 28 days, **p < 0.01 U87 versus U87 + YFSS; ^##^ *p* < 0.01 PDGC versus PDGC + YFSS, Bar = 2 mm. (F) Representative immunofluorescence images of brain sections from mice allografted with U87 or PDGC with or without YFSS sustained release stained for NEUN and labelled with Hoechst33258 at 28 dpi (left), Bar = 2 mm. The images on the right are the boxed regions in a higher magnification, Bar = 200 μm.

Our results showed that while the control groups (U87 and PDGC) exhibited rapid tumour growth, the YFSS‐treated groups (U87 + YFSS and PDGC + YFSS) demonstrated significantly slower tumour progression (Figure 7A,B). Survival analysis post‐cell transplantation indicated that YFSS treatment notably improved survival times in the mouse model: approximately 42 days for U87 + YFSS and 37.5 days for PDGC + YFSS, compared to 30 and 26.5 days in their respective controls (Figure 7C).

Histological analysis at 28 dpi confirmed a reduction in tumour volume and an increase in NEUN expression, a neuronal marker, in the YFSS‐treated groups, suggesting successful tumour cell differentiation into neurons (Figure 7D–F). These findings highlight the potential of YFSS for in situ GBM therapy, significantly reducing tumour growth and extending survival in a severe immunodeficiency mouse model.

## 4 Discussion

GBM, a complex and aggressive brain tumour, often shows limited response to traditional surgical, radiation and chemotherapy treatments, leading to poor survival outcomes. Differentiation therapy, a promising new approach for GBM, encourages tumour cells to undergo terminal differentiation, resulting in the cessation of proliferation and subsequent attenuation of tumour expansion and dissemination, while minimising deleterious effects on healthy tissues [4]. Although tumours generally have limited differentiation capacity, they hold latent potential for terminal differentiation [26]. Notably, the level of differentiation in tumours is inversely related to their malignancy [27, 28]. This novel therapeutic strategy could, therefore, represent a significant advancement in the treatment of GBM, potentially improving patient prognosis by targeting the tumour's inherent properties.

Tumour cell differentiation is also impacted by the originating tissue type. Recent studies demonstrate that combining MEK inhibitors with anti‐diabetic drugs can induce differentiation in breast cancer cells, transforming them into functional adipocytes and consequently reducing metastasis [29]. Similarly, differentiation therapy has shown promise in prostate cancer, where inducing the cells to become intermediate differentiated cells, akin to normal prostate glandular cells, effectively diminishes their malignant characteristics [30]. These findings underscore the broader applicability and potential of differentiation therapy in various cancer types, suggesting a versatile approach that adapts to the specific tissue characteristics of each tumour.

The primary focus of studies on differentiation therapy for GBM centres around the induction of differentiation into astrocytes or neurons, typically accomplished through the utilisation of chemical inducers, protein kinase inhibitors, transcription factors or miRNA transgenes [12, 13, 15, 31, 32]. Neuronal differentiation is, particularly, advantageous over astrocytic differentiation, as neurons do not proliferate, thereby aiding in halting the cell cycle. This approach is promising in improving GBM prognosis and reducing recurrence. Additionally, the use of small molecules in differentiation therapy provides significant benefits. These compounds can be synthesised and standardised easily, are cost‐effective for broad clinical use and have efficient and rapid bioavailability. This positions small molecule‐mediated therapy as a practical and potentially effective treatment strategy in the battle against GBM.

In our study, we utilised the neuron‐specific promoter *TUBB3*::mcherry to identify small molecule combinations that effectively induce neuronal differentiation in human GBM cells. The combination of Y27632, Forskolin, SB431542 and SP600125, collectively termed YFSS, proved to be efficient and rapid in driving this transformation. Live‐cell imaging revealed that YFSS induced significant morphological changes within just 2 h post‐treatment and TUBB3 expression notably increased after 3 days. Additionally, a decrease in the proliferation marker Ki‐67 and a reduction in EdU‐labelled cells were observed, alongside changes in cell cycle‐related protein expression.

After 7 days, the cells exhibited a mature neuronal phenotype, characterised by synaptic protein expression and neuron‐specific calcium transients. Transcriptome analysis and TH staining suggest these cells differentiated into dopaminergic neurons. However, it is important to note that different GBM subtypes may vary in their sensitivity to differentiation signals [33], underscoring the need to tailor differentiation strategies accordingly.

YFSS was effective across various malignant GBM cell lines and primary GBM cells from patients, achieving a remarkable conversion into neuron‐like cells and eliminating malignancy traits such as migration, invasion and stemness. These findings highlight the potential of YFSS as a versatile and potent agent for inducing neuronal differentiation in GBM, offering a promising avenue for treatment.

Extensive research has shown that modulating various signalling pathways, including cAMP, Wnt, Notch, MAPKs, mTOR, ROCK, TGF‐β and BMP, can facilitate the reprogramming of neurons. For example, combining Forskolin, a cAMP activator, with the Wnt agonist CHIR99021 has successfully induced neuron differentiation in C6 cells [12]. Similarly, adding the Notch inhibitor DAPT, the neural differentiation inducer ISX9 and I‐BET151 has led to the differentiation of human GBM cells into an immature postmitotic neuronal state [13]. The small molecule PD0325901 has effectively blocked MAPKs/ERK1/2 signalling, promoting the differentiation of GBM cells into neuron‐like cells [34]. Additionally, inhibiting MAPKs/JNK or mTOR and ROCK signalling pathways has been shown to induce differentiation into neuron‐like and astrocyte‐like cells [15, 35].

Particularly noteworthy is the role of the TGF‐β signalling pathway, which is persistently active in GBM, contributing to its malignancy and impacting neural stem cell development and differentiation [36, 37, 38, 39]. Inhibiting TGF‐β signalling has thus become a promising therapeutic strategy for GBM, employed in various differentiation protocols [40, 41, 42].

In our study, we demonstrated rapid and efficient differentiation of human GBM cells into neuron‐like cells by activating cAMP and simultaneously inhibiting ROCK, TGF‐β and MAPKs/JNK signalling pathways. This strategy significantly minimises the possibility of cells re‐entering the cell cycle and relapsing. Transcriptome analysis revealed upregulation of the Wnt pathway and downregulation of MAPK signalling during YFSS‐induced differentiation. Furthermore, we identified the inhibition of the Hippo signalling pathway as a critical factor for YFSS‐induced neural differentiation in GBM. These findings highlight the complexity of signalling pathways involved in GBM differentiation and underscore the potential of targeted manipulation of these pathways in developing effective GBM treatments.

The regulatory factor CEND1, known for its role in inhibiting cell proliferation and fostering neuronal differentiation in GBM [43], showed a consistent increase in expression during differentiation induced by YFSS. This upregulation points to CEND1's involvement in suppressing GBM cell proliferation, facilitating cell cycle exit and steering cells towards neuronal reprogramming. The partial inhibition of differentiation upon CEND1 knockdown suggests that while CEND1 is a key player, other downstream target genes are likely involved in this complex differentiation process. This finding underscores the multifaceted nature of cellular differentiation in GBM and highlights the importance of exploring additional regulatory factors that contribute to this phenomenon.

Increasing evidence indicates that cell cycle regulators, epigenetic enzymes and metabolic reprogramming factors are crucial in controlling GBM proliferation and differentiation. Our transcriptome analysis, aligned with existing research, has identified several key candidates, as shown in Figure S8. Notably, all DNA helicase MCM subtypes, typically upregulated in tumours including GBM, were consistently downregulated during differentiation, with MCM7 deficiency known to inhibit GBM proliferation [44]. Similarly, the serine/threonine protein kinase PLK1, usually promoting cell proliferation in the G2/M phase and prevalent in various tumours, showed decreased expression during differentiation, suggesting its role in cell cycle arrest and increased radiosensitivity in GBM [45].

Moreover, dual‐specificity tyrosine phosphorylation‐regulated kinase 1B (DYRK1B), associated with cell cycle exit and neuronal differentiation, was upregulated during this process. This finding aligns with previous studies showing its role in phosphorylating CyclinD1 in neuroblastoma cells [46]. In the p21‐activated protein kinase (PAK) family, we observed a decrease in oncogenic PAK1 expression, while the neuronal marker PAK3, positively correlated with GBM patient survival [47], was upregulated, indicating its potential as a therapeutic target.

The transcriptional repressor FOXG1, known to correlate with GBM progression and regulate epigenetic factors like DNMT, showed decreased expression, along with its downstream target DNMT1. This trend highlights its role in maintaining tumour stemness [48] and suggests a shift away from malignancy during differentiation. Additionally, the RNA‐binding protein SERBP1 and the dual‐specificity phosphatase DUSP1, both implicated in GBM proliferation and differentiation [49, 50], showed significant expression changes during neuronal differentiation induced by YFSS.

These findings emphasise the intricate network of molecular players in GBM differentiation, offering valuable insights into potential therapeutic targets for GBM treatment.

The ongoing evolution of reprogramming technologies and the in‐depth analysis of developmental biology data are revealing the true potential of tumour differentiation therapy. Traditional cytotoxic chemotherapy, while direct in its approach, often falls short in effectively combating human tumours. The process of transforming tumour cells into normal tissue, complex and nuanced, is becoming increasingly clear and manageable with current advancements.

However, implementing differentiation therapy in clinical settings poses several challenges. These include ensuring the efficacy and stability of induced cell differentiation, identifying various neuronal subtypes produced by different differentiation therapies and assessing their functional characteristics in vivo to evaluate their impact on normal host cells. Additionally, off‐target effects could result in neurotoxicity, immune responses or metabolic disturbances.

Efficiently delivering small molecule drugs to GBM tumours in the brain is a significant hurdle due to the restrictive nature of the blood–brain barrier (BBB), which limits the entry of many therapeutic agents. Advanced delivery strategies, such as nanoparticle‐based carriers, liposomes or convection‐enhanced delivery (CED), are necessary to ensure adequate drug penetration while minimising off‐target effects. Although micro‐osmotic pumps, used in our animal experiments, are widely utilised in preclinical research, their clinical adoption is limited due to issues like device size, lack of adjustability and regulatory requirements. Nonetheless, these devices have inspired advancements in clinical drug delivery systems, particularly, for chronic diseases and targeted therapies.

Establishing an effective and safe therapeutic window is another critical factor. Differentiation‐inducing drugs must achieve a balance between efficacy and minimal cytotoxicity to surrounding healthy brain tissue. Preclinical studies often involve dose‐escalation experiments in animal models to determine the pharmacokinetics and pharmacodynamics of the drugs. However, translating these findings to humans remains complex due to interspecies differences. In our study, we observed that YFSS sustained release in vivo at a concentration three times higher than the in vitro dosage significantly induced neural differentiation of tumour tissue with minimal toxicity.

Rigorous preclinical evaluations and clinical trials are essential to advance this approach. Trials should consider enrolling GBM patients with specific molecular subtypes that may be more responsive to differentiation therapy. Clear primary endpoints, such as progression‐free survival, overall survival and evidence of neuronal differentiation through imaging or biopsy, are crucial. Additionally, identifying the optimal combination of differentiation therapy with other treatments to enhance overall efficacy remains a critical area of focus.

In summary, inducing neuronal differentiation in GBM cells emerges as a promising therapeutic strategy. Our study provides substantial contributions to this field by offering a theoretical framework and innovative approaches for more effective and targeted GBM treatment.

## Author Contributions

**Ya'nan Hu:** conceptualisation, resources, data curation, formal analysis, supervision, funding acquisition, validation, investigation, methodology, writing‐original draft. **Jinming Liu:** conceptualisation, resources, data curation, validation, investigation. **Jian Tu:** conceptualisation, resources, data curation, validation, investigation. **Min Yang:** data curation, formal analysis, investigation, visualisation, methodology. **Qisheng He:** data curation, formal analysis, investigation, visualisation, methodology. **Fei Li:** data curation, formal analysis, investigation, visualisation, methodology. **Xiaojing Xu:** data curation, formal analysis, visualisation. **Zhongqing Ji:** data curation, formal analysis, visualisation. **Jianwei Xu:** data curation, formal analysis, visualisation. **Wentao Zhong:** formal analysis, visualisation. **Mengwen Yan:** formal analysis, visualisation. **Ying Yang:** formal analysis, visualisation. **Huanxiang Zhang:** conceptualisation, supervision, funding acquisition, methodology, project administration, writing‐review and editing. All the authors reviewed and approved the final manuscript.

## Conflicts of Interest

The authors declare no conflicts of interest.

## Supporting information


**Data S1.** Supporting Information.

**Movie S1.** Time‐lapse photography at 5‐min intervals for 5 h was conducted to observe the cell behaviour and morphological changes in U87 cells after induction with YFSS.

**Movie S2.** Live cell calcium imaging in 7 day‐differentiated U87 cells.

**Movie S3.** Live cell calcium imaging in 7 day‐differentiated U251 cells.

## Data Availability

The data that support the findings of this study are available from the corresponding author upon reasonable request.
