# Supplementary material for: Rapid and Effective Neuronal Conversion of Human Glioblastoma In Vitro and In Vivo Using Potent Small Molecules
Source: Cell Prolif. 2025 Mar 11;58(8):e70013. doi: 10.1111/cpr.70013 (PMC12336457; doi:10.1111/cpr.70013)
Supplement: Supplementary file 1 — Data S1. Supporting Information. [file CPR-58-e70013-s003.docx]

**Supplementary Figures**

**
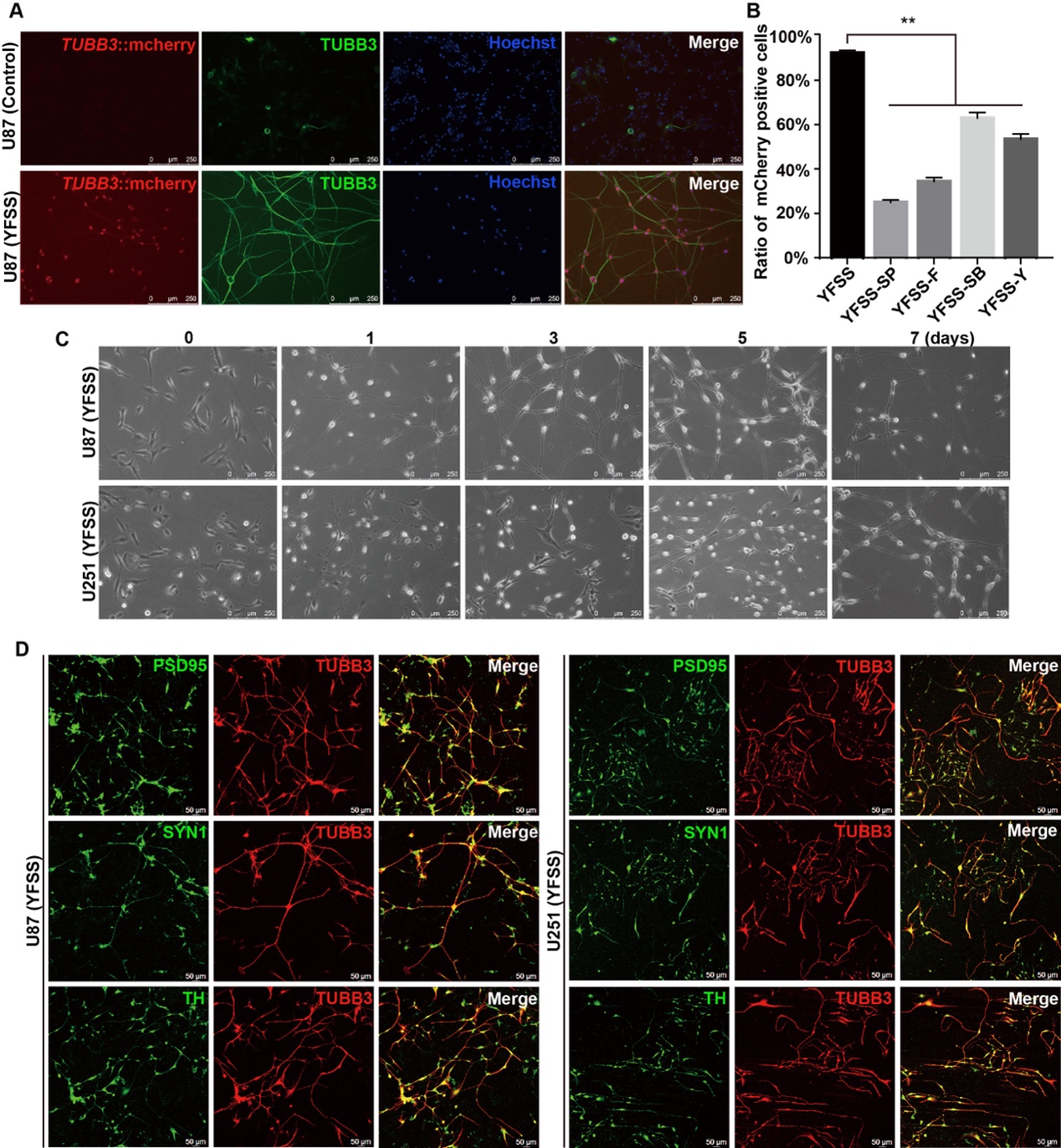
**

**Figure S1.** Screening to identify a small molecule combination that effectively induces the differentiation of GBM cells into neuron-like cells. **(A)** Representative images of U87 modified with a *TUBB3* promoter-reporter system (*TUBB3*::mcherry) treated with YFSS for 7 days and stained for TUBB3 and labeled with Hoechst33258, Bar=250 μm. **(B)** Reduction of either component led to a decrease in the percentage of mCherry-positive cells in U87, ***P*<0.01. **(C)** Representative images of morphological changes of YFSS-induced GBM cells at different stages (0, 1, 3, 5, and 7 days), Bar=250 μm. **(D)** Representative images of GBM cells treated with YFSS for 7 days double stained for TUBB3 and PSD95, SYN1, or dopaminergic neuronal marker TH, Bar=50 μm.

**
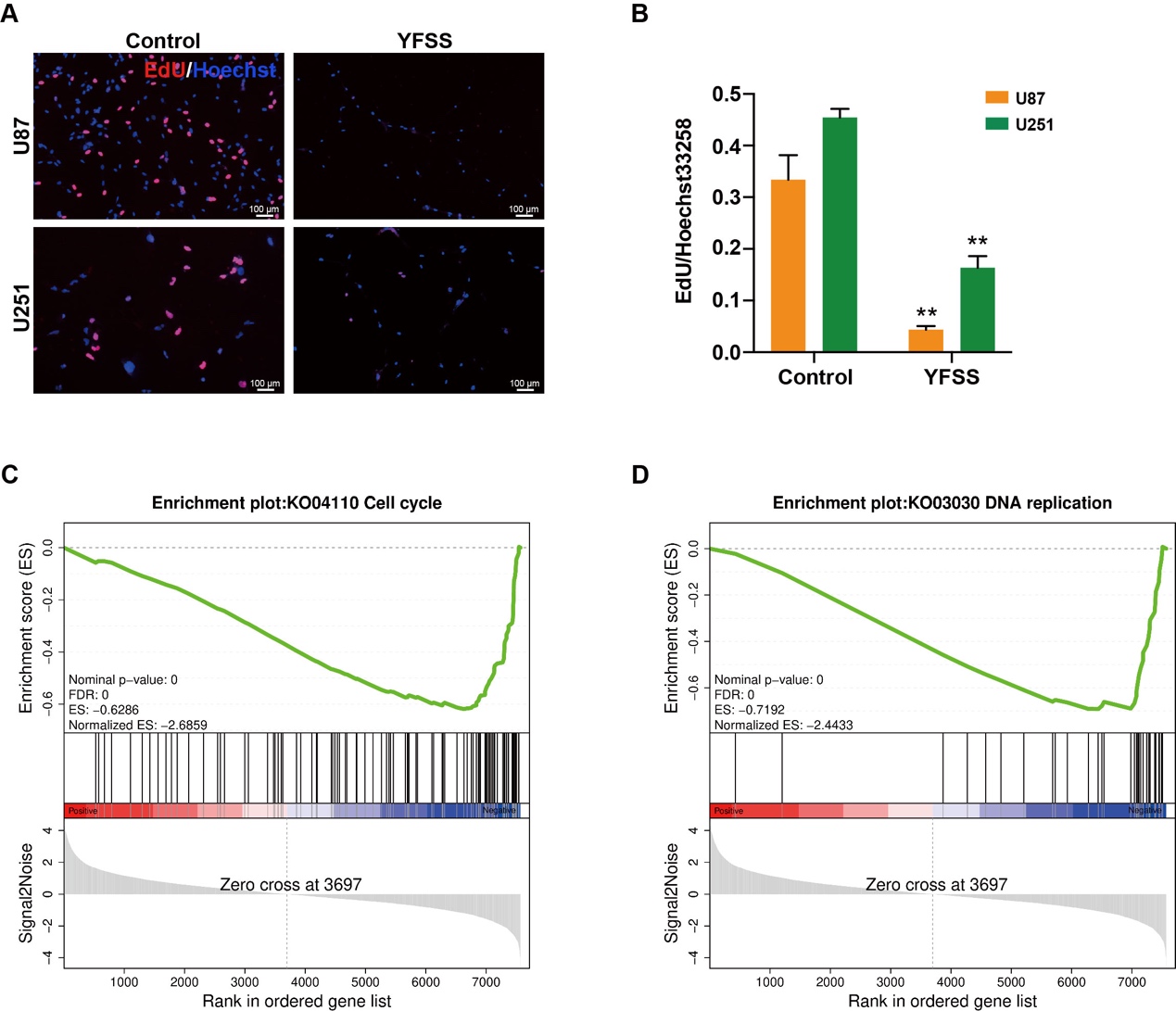
Figure S2. (A** and **B)** Representative images of GBM cells treated with YFSS for 7 days labeled with EdU and Hoechst33258 **(A)** and the relative EdU incorporation rate was calculated **(B)**, ***P*<0.01, Bar=100 μm. **(C** and **D)** GSEA graphs for two enriched pathways, cell cycle and DNA replication (Day 3 vs Day 0).

**
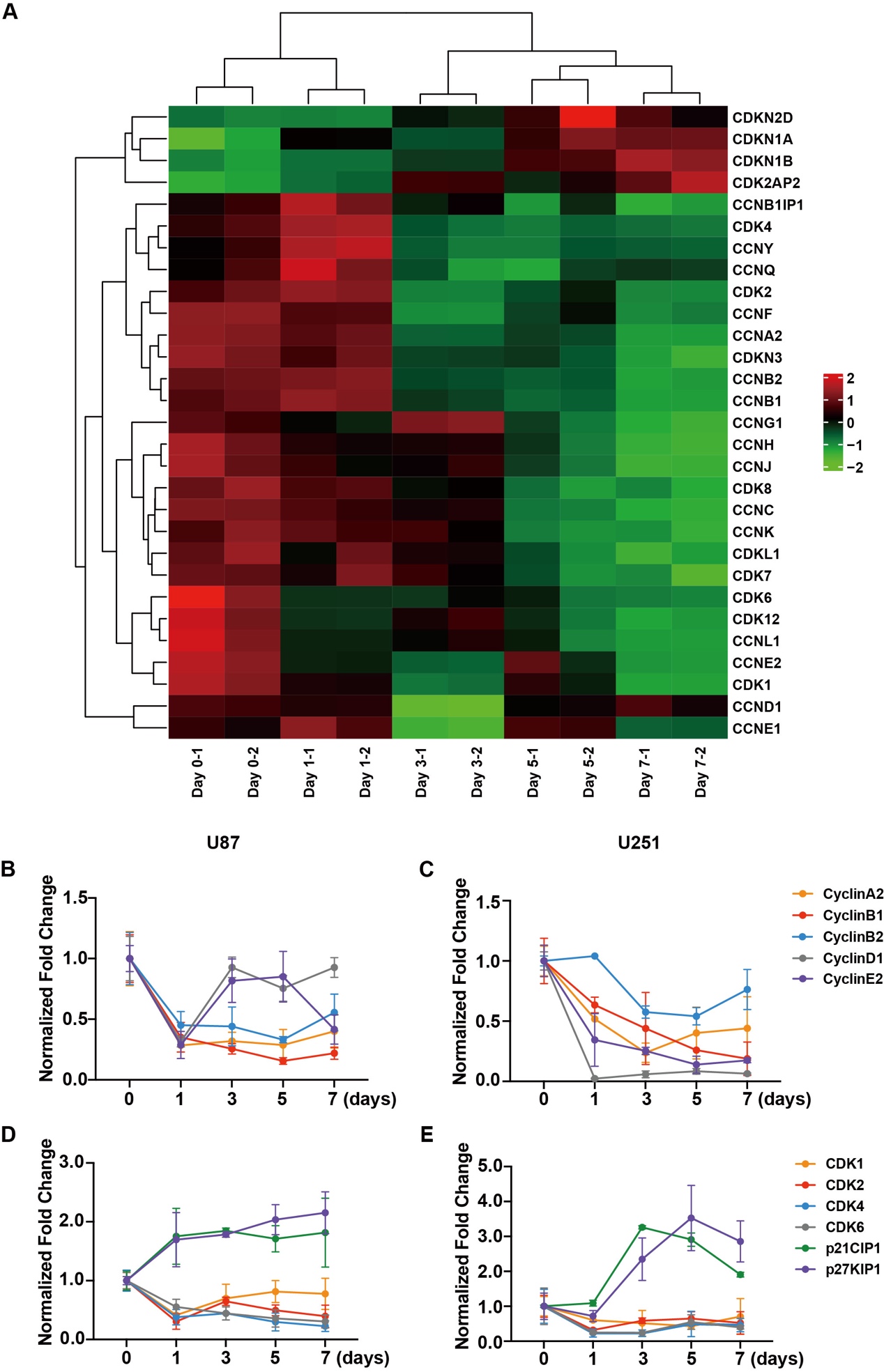
**

**Figure S3.** Changes in the expression pattern of cyclin genes occur during YFSS-induced GBM differentiation. **(A)** Heatmap of CKIs, Cyclins, and CDKs expression in transcriptomic data. **(B-E)** qRT-PCR of Cyclins, CDKs, and CKIs expression in YFSS-induced GBM cells at different stages (0, 1, 3, 5, and 7 days).

**
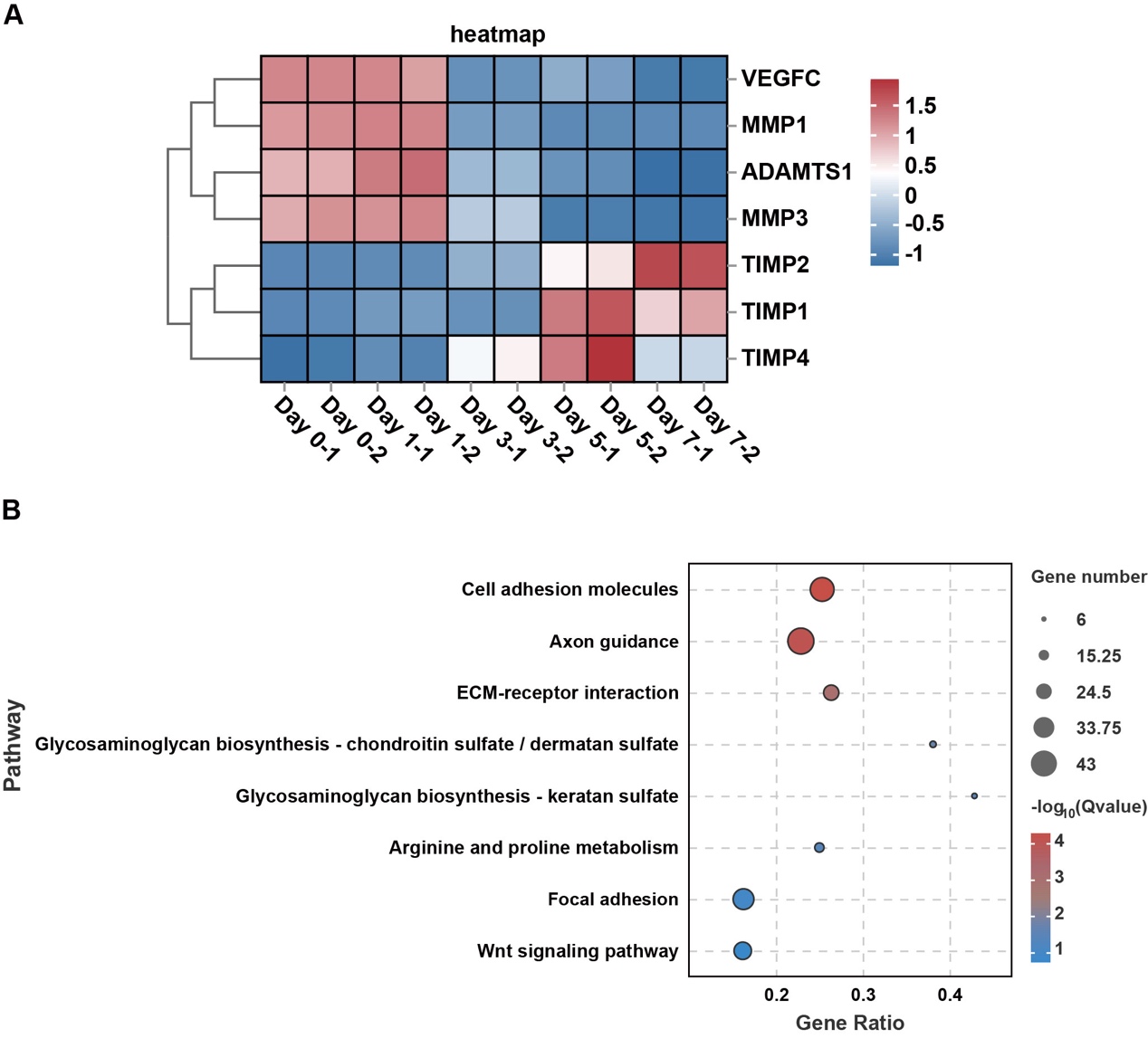
**

**Figure S4. (A)** Heatmap of invasion-related gene expression in transcriptomic data. **(B)** KEGG analysis in the pathway of the key modules (19, consistent increase) from WGCNA.

**
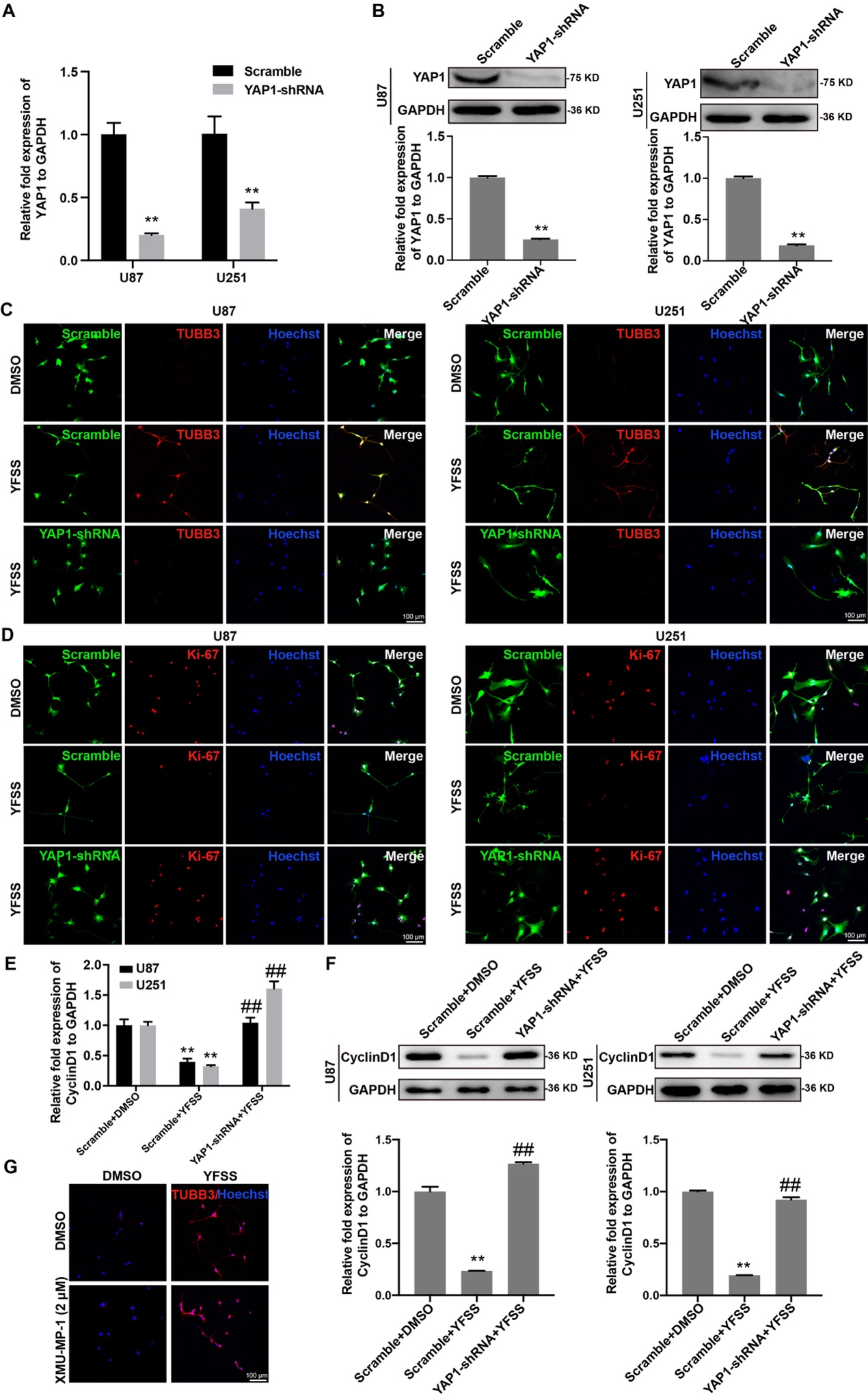
**

**Figure S5. (A** and **B)** Relative expression of YAP1 in U87 and U251 cells treated with control siRNA (Scramble) or siRNA targeting YAP1 (YAP1-shRNA) by qRT-PCR analysis **(A)** and Western blot analysis **(B)**, ***P*<0.01. **(C** and **D)** Representative images of GBM cells treated with control siRNA (Scramble) or siRNA targeting YAP1 (YAP1-shRNA) induced by YFSS for 3 days and stained for TUBB3 **(C)** and Ki-67 **(D)**, and labeled with Hoechst33258, Bar=100 μm. **(E** and **F)** Relative expression of CyclinD1 in U87 and U251 cells treated with control siRNA (Scramble) or siRNA targeting YAP1 (YAP1-shRNA) induced by YFSS for 1 day by qRT-PCR analysis **(E)** and Western blot analysis **(F)**, ***P*<0.01 vs. Scramble; ^##^*P*<0.01 vs. Scramble+YFSS, **(G)** Representative images of GBM cells treated with DMSO, YFSS and/or XMU-MP-1 (2 μΜ) for 7 days, stained for TUBB3 and labeled with Hoechst33258, Bar=100 μm.

**
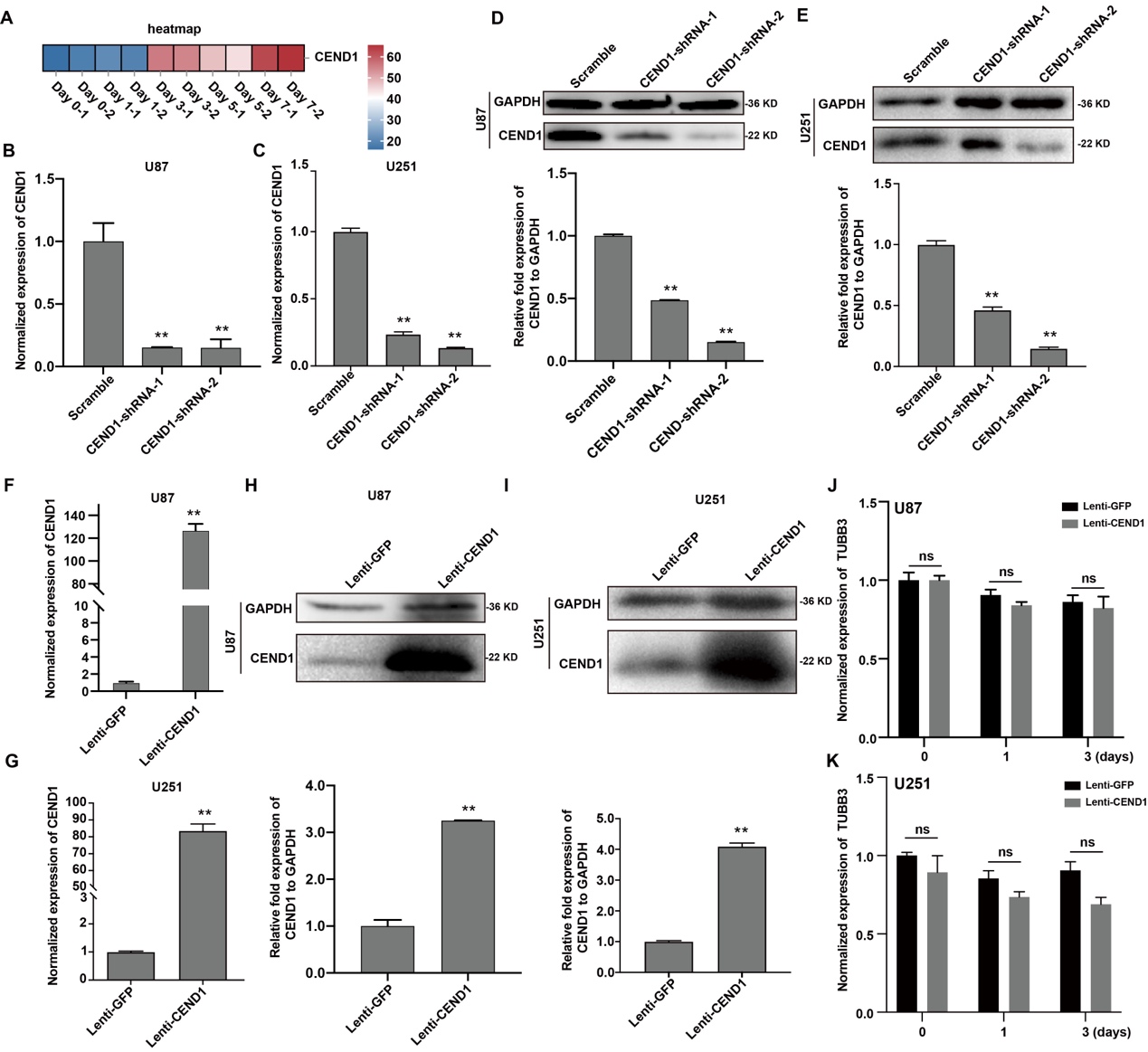
**

**Figure S6. (A)** Relative expression of CEND1 in YFSS-induced GBM cells at different stages (0, 1, 3, 5, and 7 days) in transcriptomic data. **(B-E)** Relative expression of CEND1 in U87 and U251 cells treated with control siRNA (Scramble) or siRNA targeting CEND1 (CEND1-shRNA-1 or CEND1-shRNA-2) by qRT-PCR analysis **(B** and **C)** and Western blot analysis **(D** and **E)**, ***P*<0.01. **(F-I)** Relative expression of CEND1 in U87 and U251 cells of control (Lenti-GFP) or overexpressed CEND1 (Lenti-CEND1) by qRT-PCR analysis **(F** and **G)** and Western blot analysis **(H** and **I)**, ***P*<0.01. **(J** and **K)** qRT-PCR of TUBB3 expression in GBM cells (U87 and U251) of control (Lenti-GFP) or overexpressed CEND1 (Lenti-CEND1) for different times (0, 1, and 3 days), ns, not significant, *P*>0.05.

**
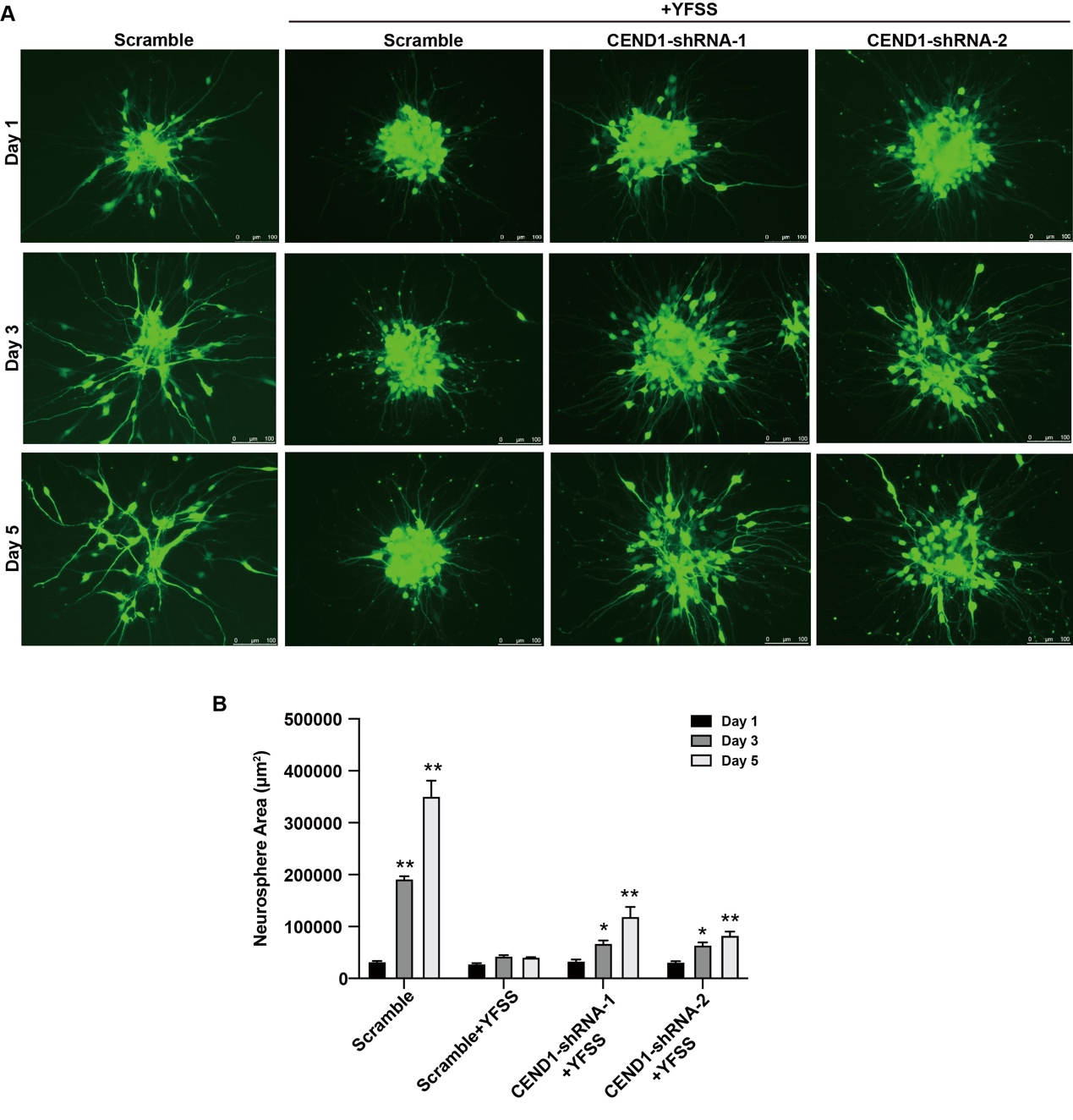
**

**Figure S7. (A** and **B)** Representative images from PDGC with indicated treatments for different time points (1, 3, and 5 days) **(A)**, and statistical analysis of average area of tumor spheres **(B)**, **P*<0.05, ** *P*<0.01 vs. Day 1, Bar=100 μm.

**
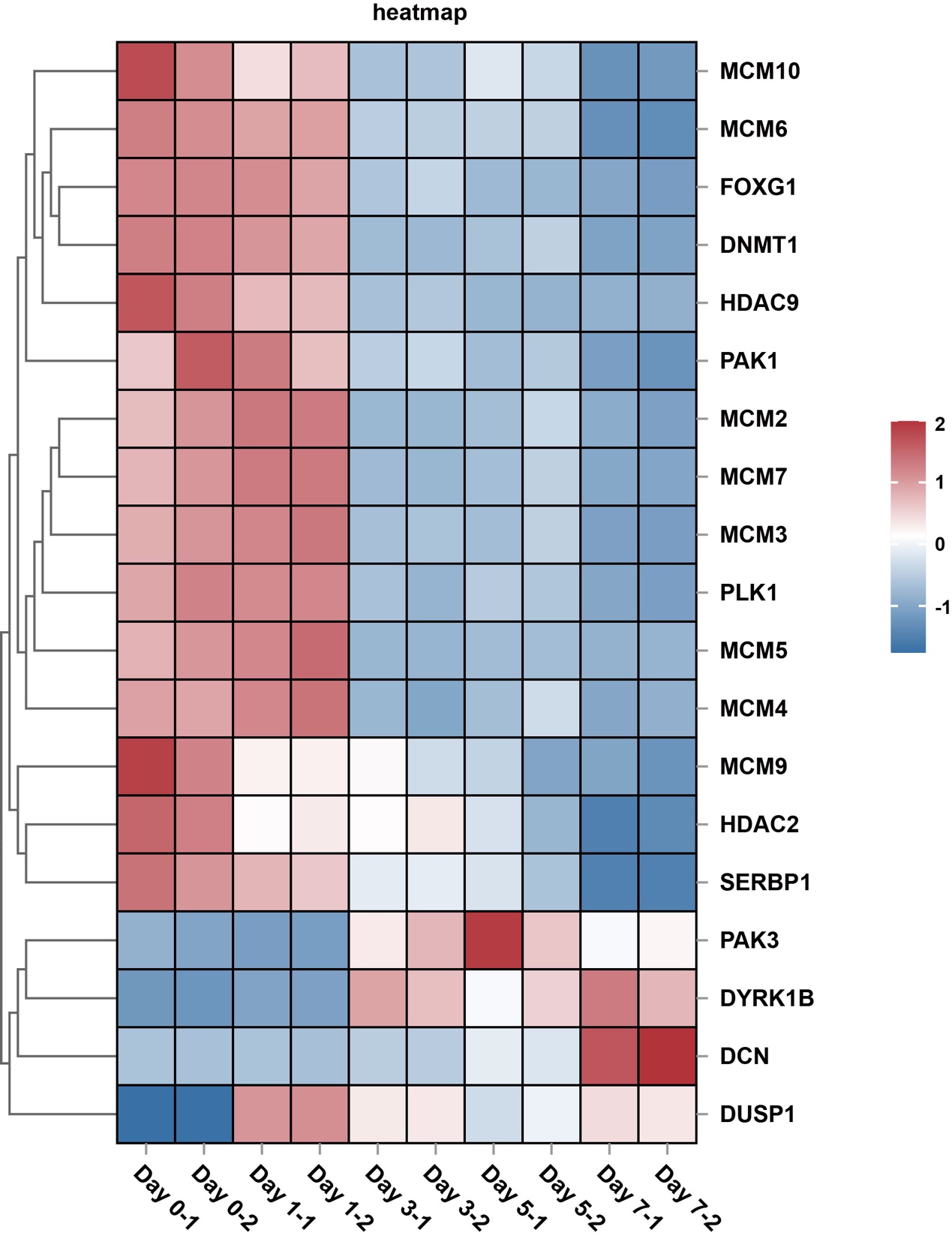
**

**Figure S8.** A heatmap of candidate gene expression in transcriptomic data

| genes | Sense | Anti-Sense |
| --- | --- | --- |
| CDK1 | GGATGTGCTTATGCAGGATTCC | CATGTACTGACCAGGAGGGATAG |
| CDK2 | CCAGGAGTTACTTCTATGCCTGA | TTCATCCAGGGGAGGTACAAC |
| CDK4 | ATGGCTACCTCTCGATATGAGC | CATTGGGGACTCTCACACTCT |
| CDK6 | CCAGATGGCTCTAACCTCAGT | AACTTCCACGAAAAAGAGGCTT |
| CEND1 | CCAAGCCCTCGAAGAAGGAG | CTTCAGGTTGCTGTGGTTGT |
| CyclinA2 | GGATGGTAGTTTTGAGTCACCAC | CACGAGGATAGCTCTCATACTGT |
| CyclinB1 | TTGGGGACATTGGTAACAAAGTC | ATAGGCTCAGGCGAAAGTTTTT |
| CyclinB2 | TGCTCTGCAAAATCGAGGACA | GCCAATCCACTAGGATGGCA |
| CyclinD1 | GCTGCGAAGTGGAAACCATC | CCTCCTTCTGCACACATTTGAA |
| CyclinE2 | GGAACCACAGATGAGGTCCAT | CCATCAGTGACGTAAGCAAACT |
| GAPDH | AACATCATCCCTGCCTCTACTGG | GTTTTTCTAGACGGCAGGTCAGG |
| Ki-67 | ACGCCTGGTTACTATCAAAAGG | CAGACCCATTTACTTGTGTTGGA |
| MAP2 | CAGGAGACAGAGATGAGAATTCC | CAGGAGTGATGGCAGTAGAC |
| MMP1 | CTCTGGAGTAATGTCACACCTCT | TGTTGGTCCACCTTTCATCTTC |
| MMP3 | CGGTTCCGCCTGTCTCAAG | CGCCAAAAGTGCCTGTCTT |
| NEUN | TCGTAGAGGGACGGAAAATTGA | GCCGTTGGTGTAGGGGTTC |
| p21CIP1 | CGATGGAACTTCGACTTTGTCA | GCACAAGGGTACAAGACAGTG |
| p27KIP1 | AACGTGCGAGTGTCTAACGG | CCCTCTAGGGGTTTGTGATTCT |
| TUBB3 | ATGTCCTCCACCTTCATC | GAACTCCATCTCGTCCAT |
| Scramble for CEND1 | ACGACGATGCGAGGACGCGAT |  |
| CEND1-shRNA-1 | GGAGGACGCGCCCATAGAATG |  |
| CEND1-shRNA-2 | GAGTCTAACCAGCGTATAATG |  |
| CEND1  (PCR) | CGGAATTCATGGAGTCCAGAGGGAAGTCAG | CGGGATCCTTATTTTTTCCGGACCAGGAAG |
| Scramble for YAP1 | AGTTCGATTACAATCCGTCCA |  |
| YAP1-shRNA | GACCAATAGCTCAGATCCTTT |  |
| YAP1  (qRT-PCR) | TAGCCCTGCGTAGCCAGTTA | TCATGCTTAGTCCACTGTCTGT |

**Table S1:** The sequence of primers

**Movie S1:**

Time-lapse photography at 5-minute intervals for 5 hours was conducted to observe the cell behavior and morphological changes in U87 cells after induction with YFSS.

**Movie S2:**

Live cell calcium imaging in 7 day-differentiated U87 cells

**Movie S3:**

Live cell calcium imaging in 7 day-differentiated U251 cells
